# Identification of Keratinocyte Growth Factor as a Target of microRNA-155 in Lung Fibroblasts: Implication in Epithelial-Mesenchymal Interactions

**DOI:** 10.1371/journal.pone.0006718

**Published:** 2009-08-24

**Authors:** Nicolas Pottier, Thomas Maurin, Benoit Chevalier, Marie-Pierre Puisségur, Kevin Lebrigand, Karine Robbe-Sermesant, Thomas Bertero, Christian L. Lino Cardenas, Elisabeth Courcot, Géraldine Rios, Sandra Fourre, Jean-Marc Lo-Guidice, Brice Marcet, Bruno Cardinaud, Pascal Barbry, Bernard Mari

**Affiliations:** 1 CNRS, Institut de Pharmacologie Moléculaire et Cellulaire, UMR6097, Sophia Antipolis, France; 2 University of Nice Sophia-Antipolis, Nice, France; 3 EA2679, Faculté de Médecine H. Warembourg, Pôle Recherche, Lille, France; University of Hong Kong, Hong Kong

## Abstract

**Background:**

Epithelial-mesenchymal interactions are critical in regulating many aspects of vertebrate embryo development, and for the maintenance of homeostatic equilibrium in adult tissues. The interactions between epithelium and mesenchyme are believed to be mediated by paracrine signals such as cytokines and extracellular matrix components secreted from fibroblasts that affect adjacent epithelia. In this study, we sought to identify the repertoire of microRNAs (miRNAs) in normal lung human fibroblasts and their potential regulation by the cytokines TNF-α, IL-1β and TGF-β.

**Methodology/Principal Findings:**

MiR-155 was significantly induced by inflammatory cytokines TNF-α and IL-1β while it was down-regulated by TGF-β. Ectopic expression of miR-155 in human fibroblasts induced modulation of a large set of genes related to “cell to cell signalling”, “cell morphology” and “cellular movement”. This was consistent with an induction of caspase-3 activity and with an increase in cell migration in fibroblasts tranfected with miR-155. Using different miRNA bioinformatic target prediction tools, we found a specific enrichment for miR-155 predicted targets among the population of down-regulated transcripts. Among fibroblast-selective targets, one interesting hit was keratinocyte growth factor (KGF, FGF-7), a member of the fibroblast growth factor (FGF) family, which owns two potential binding sites for miR-155 in its 3′-UTR. Luciferase assays experimentally validated that miR-155 can efficiently target KGF 3′-UTR. Site-directed mutagenesis revealed that only one out of the 2 potential sites was truly functional. Functional *in vitro* assays experimentally validated that miR-155 can efficiently target KGF 3′-UTR. Furthermore, *in vivo* experiments using a mouse model of lung fibrosis showed that miR-155 expression level was correlated with the degree of lung fibrosis.

**Conclusions/Significance:**

Our results strongly suggest a physiological function of miR-155 in lung fibroblasts. Altogether, this study implicates this miRNA in the regulation by mesenchymal cells of surrounding lung epithelium, making it a potential key player during tissue injury.

## Introduction

Epithelial-mesenchymal interactions are critical in regulating many aspects of vertebrate embryo development and for the maintenance of homeostatic equilibrium in adult tissues [1]. Stromal cells maintain control over epithelial cell proliferation, survival and response to wounds and could also play a collaborative role in pathological conditions such as cancer [2]. The interactions between epithelium and mesenchyme are believed to be mediated by paracrine signals and extracellular matrix (ECM) components secreted from fibroblasts thereby affecting adjacent epithelia. In response to paracrine signals such as cytokines and direct-cell contact from adjacent tumor epithelial cells, fibroblasts undergo changes that may alter the normal epithelial-mesenchymal interactions [3]–[5].

This is in particular the case during pulmonary fibrosis, where it has been demonstrated that a variety of cytokines such as transforming growth factor-beta (TGF-β), tumor necrosis factor-alpha (TNF-α), fibroblast growth factors (FGFs), interleukin-1 (IL-1), monocyte chemoattractant protein-1 (MCP-1) are produced at the sites of active fibrosis. These cytokines appear to be mostly expressed by activated inflammatory cells, such as macrophages and eosinophils [6]. A balance of pro-fibrogenic and anti-fibrogenic forces generated by these networks of cytokines determines the outcome of lung injury and inflammation. *In vitro* studies of individual cytokines on fibroblasts has revealed a variety of roles for these cytokines in the regulation of the fibrotic process, such as mitogenic activity, stimulation of extracellular matrix and alpha-smooth muscle actin gene expression, alteration of the contractile phenotype, production of proteases, protease inhibitors, chemokines, cytokines and growth factors [7].

Recently, microRNAs (miRNAs) have emerged as a major class of gene expression regulators linked to many biological functions. Since the first miRNA was identified in *Caenorhabditis elegans* as an important factor for timing of larval development [8], [9], thousands of miRNAs have been characterized including about 700 miRNAs in the human genome [10]. MiRNAs are derived from a primary transcript called pri-miRNAs. The current model of maturation includes primary nuclear cleavage of pri-miRNAs by the RNase III endonuclease Drosha, which liberates pre-miRNA hairpins. Hairpins are exported from the nucleus to the cytoplasm, where they are cleaved by Dicer, another RNase III endonuclease [11]–[13]. Dicer generates short RNA sequences of about 22-nucleotides. MiRNAs are then assembled with proteins of the Argonaute family into a ribonucleoprotein complex: miRNP [14], which exhibits binding complementarities with sequences usually located in the 3′UTR of the target transcript. The formation of a complex between miRNP and a target leads to a repression of protein synthesis, often associated with mRNA degradation [15]. Complex formation follows a set of rules that have been substantiated by experimental evidences [16]–[18]. The major determinant of the interaction between the miRNA and its targets corresponds to a short stretch of 6–8 nucleotides located 5′ of the miRNA, called the “seed”. This implies that one miRNA can theoretically interacts with hundreds of mRNAs [19]. Because several miRNAs can also target the same transcript, the miRNA regulatory network appears amazingly complex. To date, a small proportion of miRNA targets have been validated and the exact function of most miRNAs remains to be elucidated.

Dysregulation of these molecules, including miR-155 [20], has been recently identified in the pathogenesis of inflammatory diseases [20]. MiR-155 is contained within the only phylogenetically conserved region of BIC RNA [21], [22]. It has been linked to cancer [23]–[26], viral infection [27] and immunity [28]–[31]. MiR-155 has been shown to be induced by proinflammatory stimuli such as lipopolysaccharides (LPS), Toll-like receptors (TLRs), IL-1 and TNF-α in macrophages and dendritic cells [32]–[34]. It has also been detected in synovial fibroblasts and rheumatoid synovial tissue [35]. Multiple targets for miR-155 have been identified in several cell types and linked with the regulation of B and T cell differentiation [29], [30], [36]–[38], TLR signalling in inflammatory cells [34], or cellular adhesion in epithelial malignancies [39]. To date, the putative function of miR-155 in fibroblasts remains poorly documented [35], [40].

The aim of the present study was to identify the repertoire of miRNA expressed in fibroblasts and to characterize their potential regulation by inflammatory cytokines. The detection of miR-155 into these cells led us to investigate its relationship with keratinocyte growth factor (KGF, FGF7), a central factor in tissue repair that we are establishing as a direct target of miR-155.

## Results

### miRNA expressed in normal lung fibroblast cells and their modulation by inflammatory cytokines

**Figure 1:**
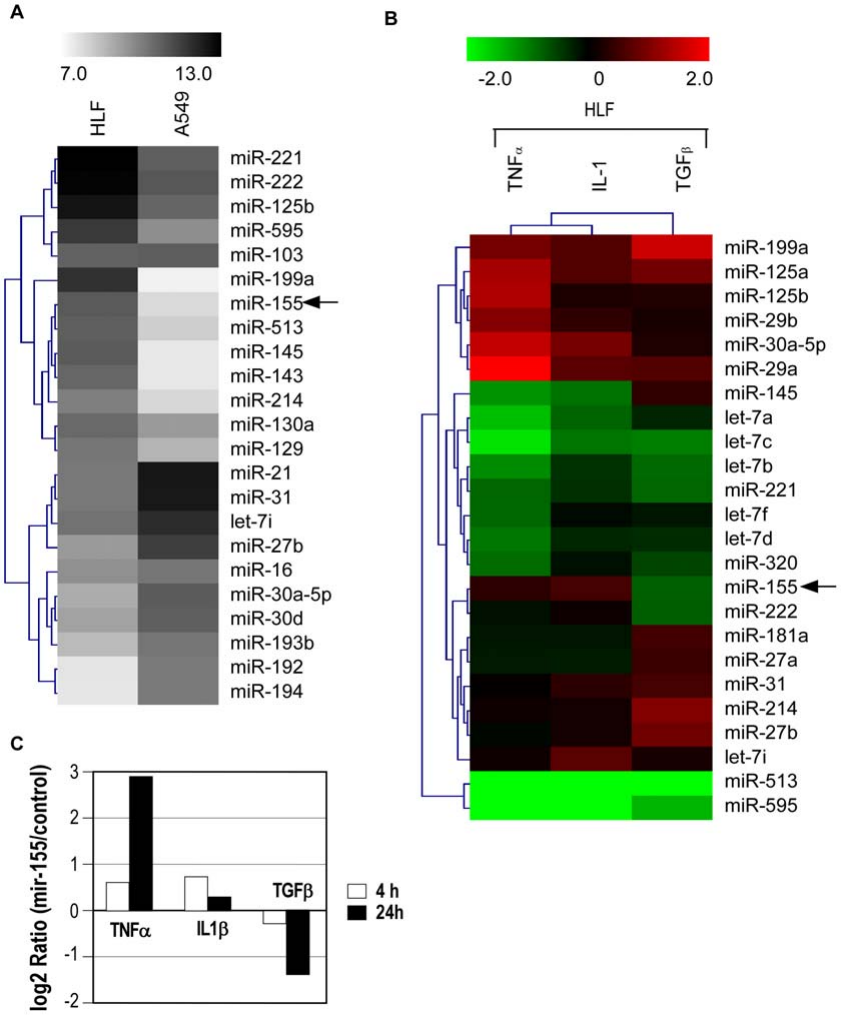
MiRNA expression data in human adenocarcinoma cells A549 and normal lung fibroblasts (CCD-19Lu) following a 24 h stimulation with TNF-α, IL-1β or TGF-β. Cells were stimulated by the different cytokines and small RNA fraction from different biological conditions were chemically labelled and hybridized on a miRNA microarray as described in the Materials and Methods section. (A) Heatmap comparing control human pulmonary fibroblasts versus A549 adenocarcinoma cells. (B) Hierarchical clustering of miRNA modulated in normal human pulmonary fibroblasts following stimulation with 10 ng/ml TNF-α, IL-1β and TGF-β. (C) Analysis of miR-155 expression levels by Taqman miRNA assay following a 4 or 24 h stimulation by the 3 cytokines.

We first analyzed the miRNA expression profile of normal human pulmonary fibroblasts (HFL1, CCL-153) and compared it with the miRNome of the alveolar epithelial cell line A549. Large differences distinguish the 2 cell lines, such as a high expression of the oncogenic miR-21 in the epithelial tumor cells or the restricted expression of miR-199a, miR-143, miR-145 and miR-155 to fibroblasts (Fig. 1A and Table S1). The detection of miR-155, a major effector of B and T cells into fibroblasts confirm previous studies indicating that this miRNA can be also detected in non haematological tissues [40], [41]. Because miR-155 has been previously shown to be upregulated by inflammatory cytokines in macrophages [32], we then analyzed the potential regulation of miR-155 by inflammatory cytokines. Results indicate that miR-155 was slightly induced by TNF-α and IL-1β while its level decreased following TGF-β stimulation (Fig. 1B). Interestingly, several other miRNAs, such as miR-29a/b, miR-30a, miR-125a/b and miR-199 varied in a similar manner following stimulation, with a milder effect of IL-1β relative to TNF-α (Fig. 1B). Levels of mature miR-155 following treatment with the three cytokines were determined by quantitative RT-PCR (Fig.1C). Cells were serum starved 2 hours before treatment and subsequently stimulated either for 4 h or 24 h. After 4 h of TNF-α and IL-1β stimulation, the level of miR-155 displayed a slight increase while it decreased in presence of TGF-β (Fig. 1C). A longer treatment led to a more pronounced effect of TNF-α and TGF-β while the level of miR-155 returned to its basal level in presence of IL1β. Of note, no significant modulation of miR-155 could be observed in A549 cells (data not shown).

### MiR-155 expression in a mouse model of lung fibrosis

**Figure 2:**
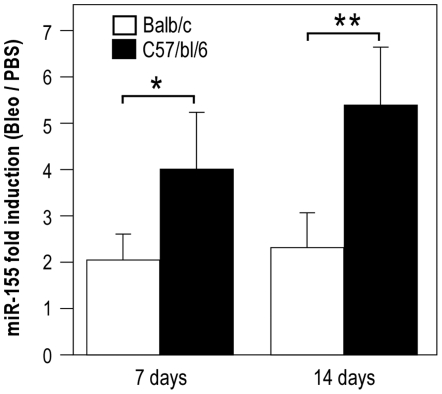
MiR-155 expression is associated with lung fibrosis in a murine model. Bleomycin-sensitive (C57BL/6) and resistant (BALB/c) mice were treated with bleomycin or PBS and killed at various times after instillation. Total RNA was extracted from lungs and miR-155 expression levels were performed by taqman miRNA assay. Histogram represents miR-155 fold induction between bleomycin and PBS for the 2 mice strains 7 and 14 days after instillation (n = 4 animals/group).

We then investigated the expression of miR-155 in a mouse model of lung fibrosis, using the well-established bleomycin instillation model. MiR-155 expression was measured by quantitative RT-PCR on lungs from either sensitive C57BL/6 or resistant BALB/c mice 7 and 14 days after bleomycin instillation and compared with a PBS control (Fig. 2). Bleomycin impact on two mouse strains was significantly different as evaluated by survival and histopathological analysis, as previously described [42]. Bleomycin led to an induction of miR-155 at both times in the two strains. Interestingly, this induction appeared significantly more pronounced in the sensitive C57BL/6 compared to the resistant BALB/c mice (Fig. 2). Overall, these data indicated that miR-155 can also be induced in an *in vivo* fibrogenic model and that its expression level was correlated with the degree of lung fibrosis.

### mRNA profiling of miR-155-overexpressing fibroblasts

**Figure 3:**
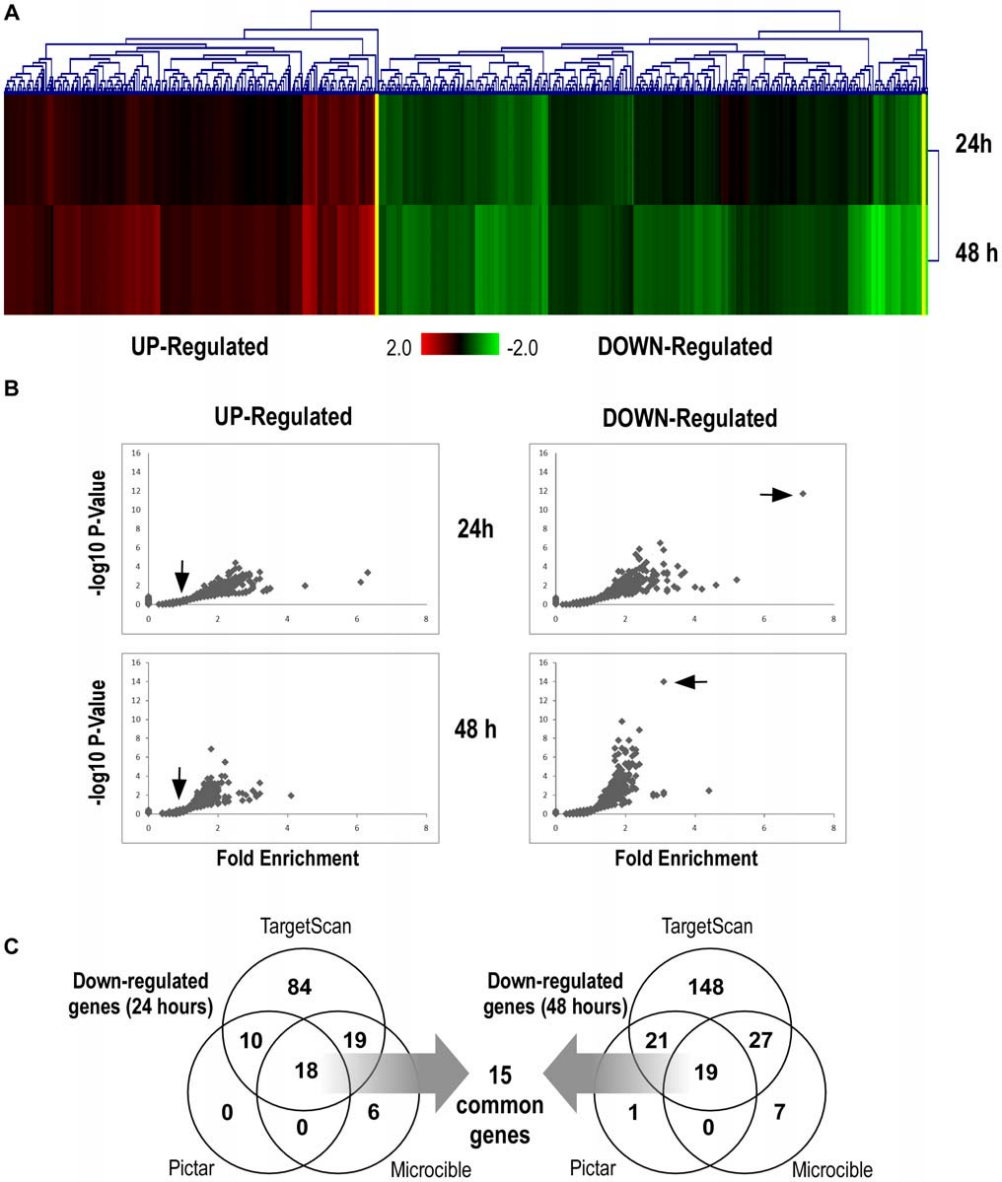
A large number of predicted targets are repressed after overexpression of miR-155. Lung fibroblasts were transfected with a synthetic pre-miR-155 or with a negative pre-miRNA (N = 2). RNA samples were harvested at 24 and 48 h post-transfection. mRNA profiles were determined with pan genomic arrays. (A) Heatmap comparing the normalized log2 of the ratios between pre-miR-155 signal and the pre-miR-Neg signal, 24 h and 48 h after transfection. Clustering was performed using a Euclidian distance and a Ward method of agglomeration. Colours vary from green for the lowest ratios to red for the highest ratios. (B) Over-representation of miR-155 seed complementary sequences in the 3′-UTR of downregulated transcripts. Using our bioinformatics tool “MicroTopTable” (see materials and method), representation of miRNA seed-binding sequences in the set of up- or down-regulated genes was compared with the set of all expressed genes. For each miRNA seed, a fold enrichment value (horizontal axis) and an associated P-Value (vertical axis) were calculated. The arrow indicates miR-155. (C) Venn diagram comparing the number of miR-155 targets among the set of downregulated genes at 24 h and 48 h post-transfection according to 3 distinct target prediction tools (TargetScan, Pictar and MicroCible).

**Figure 4:**
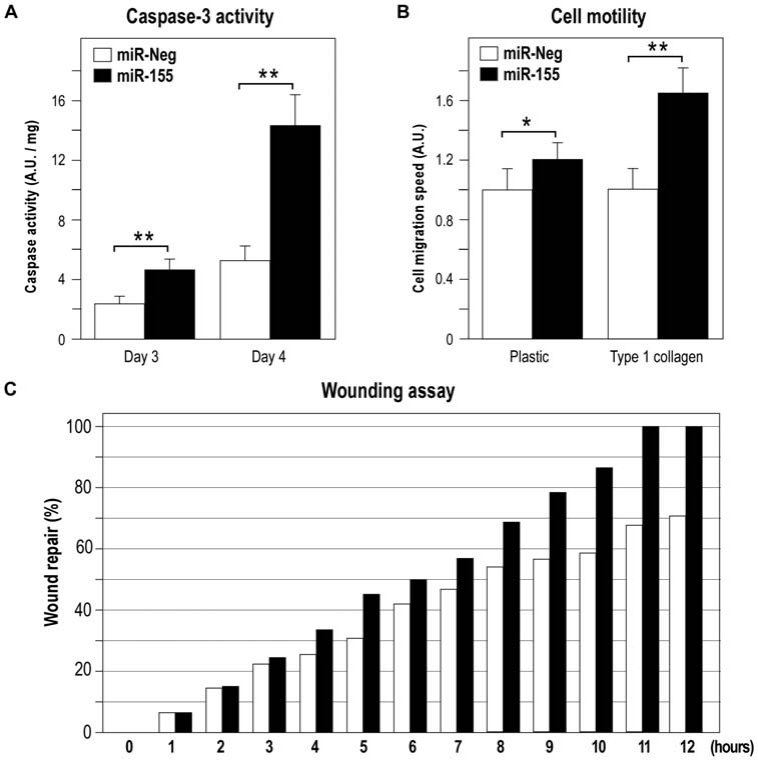
Transfection of human lung fibroblasts with pre-miR-155 increases caspase-3 activity and cell motility. Fibroblasts were transfected with either pre-miR-Neg or pre-miR-155 (10 nM). (A) Caspase 3/7 assay was carried out 3 and 4 days following transfection using a specific substrate as described in the method section (n = 3). (B) A scratch wound was induced in confluent cell monolayers plated on plastic or on type I collagen and individual cell migration speed was evaluated on 20 independant cells (n = 2). (C) Representative experiment showing the percentage of the repaired area in a scratch wound assay (n = 2).

We then looked for fibroblast-specific targets of miR-155. To that end, we transfected lung fibroblasts HFL1 either with a synthetic pre-miR-155 or a “negative” pre-miRNA, used as a control. Two independent experiments were carried out and the expression of mature miR-155 was monitored with RQ-PCR. A ∼500 fold induction of the miRNA was measured (data not shown). We then used human pan genomic arrays [43] to determine mRNA profiles and identify genes whose expression levels changed following miR-155 overexpression. The analysis was performed on RNA samples harvested at 24 h and 48 h post-transfection, as suggested by recent similar studies [29], [30], [44]. Statistical analysis revealed changes in the expression of 474 genes (197 up and 277 down-regulated) and 1404 genes (619 up and 785 dow-regulated) at 24 h and 48 h after transfection, respectively. As shown in Fig. 3A, the vast majority of modulated genes at 24 hours were also regulated at 48 hours. When the 2 sets of genes were analyzed by functional annotations, both lists were associated with similar biological functions such as “cell-to-cell signalling and interaction”, “cell death” and “cellular movement” (Table S2). Two distinct functional assays indeed confirmed these predictions: firstly, caspase-3 activity was increased in miR-155-transfected fibroblasts compared to control cells (Fig. 4A), suggesting that miR-155 could promote apoptosis; Secondly, transfection of fibroblasts with miR-155 altered their migration. Cell motility was especially increased for fibroblasts migrating on a type I-collagen substrate, for which speed migration increased to 60% of basal (Fig. 4B). In agreement with these data, analysis of scratch wound repair on a collagen type I substrate indicated that a monolayer of fibroblasts expressing mir-155 was more rapidly repaired than a monolayer of control cells (Fig. 4C).

We observed after heterologous overexpression of miR-155 the reduced level of expression of a population of transcripts that corresponded to predicted targets of miR-155. Three independent prediction tools (TargetScan, Pictar and MicroCible, see materials and method section for calculation details) indicated an over-representation of miR-155 predicted targets in the population of down-regulated transcripts. Indeed, ∼7 fold enrichment for the presence of a 8-nucleotide sequence complementary to the miR-155 “seed” was detected at 24 h (Fig. 3B). A ∼3-fold enrichment was noticed at 48 h (Fig. 3B). Different prediction tools provided contrasted results, as visualized in the Venn diagrams depicted in Fig. 2C. While a total of 260 transcripts were predicted by at least one of this algorithm (Table S3), only 18 and 19 transcripts were predicted by all 3 bioinformatics tools at 24 and 48 hours, respectively. 15 transcripts were common to the 2 lists (Table 1). Importantly, 9 out of these 15 transcripts (BACH1, H3F3A, MAP3K7IP2, RAB11FIP2, SDCBP, SGK3, TOMM20, TSPAN14, YWHAZ) have already been described as miR-155 targets in immune models [27], [29], [30], [45]. This experimental approach was also validated with the “Sylamer” approach described in [46]. Overexpression of miR-155 in fibroblasts at 24 h and at 48 h clearly led to a down-regulation of transcripts containing the following sequences in their 3′UTR: “GCATTA”, “GCATTAA”, “AGCATTA”, “AGCATTAA” (data not shown). These sequences indeed correspond to the sequences complementary to the miR-155 “seed” region. We noticed that the best miR-155 “seed” enrichment was obtained when predicted sites were selected in the 3′UTR rather than in the rest of the transcript (i.e. in the open reading frame and in the 5′UTR, data not shown).

**Table 1:** miR-155 targets specific of human lung fibroblasts as determined by a combination of microarray analysis and of computational target search.

Name	ID RNG	UNIGENE ID	ACCESSION NUMBER	DESCRIPTION	Log2 Ratio		Adj.P.Val	
					24 h	48 h	24 h	48 h
ANTXR2	49597	Hs.162963	NM_058172	Anthrax toxin receptor 2 (ANTXR2), mRNA.	−1.30	−1.54	1.49E-05	3.82E-06
BACH1	90782	Hs.154276	NM_001186;NM_206866	BTB and CNC homology 1, basic leucine zipper transcription factor 1 (BACH1), transcript variant 1, mRNA.	−0.85	−1.12	2.78E-04	4.75E-05
CSNK1A1	91649	Hs.529862	NM_001025105;NM_001892	Casein kinase 1, alpha 1 (CSNK1A1), transcript variant 2, mRNA.	−0.98	−1.29	1.53E-05	1.52E-06
FGF7	129551	Hs.567268	NM_002009	Fibroblast growth factor 7 (keratinocyte growth factor) (FGF7), mRNA.	−0.78	−1.33	2.71E-03	9.21E-05
FGF7	172697	Hs.567268	NM_002009;AK054997	cDNA FLJ30435 fis, clone BRACE2009031, weakly similar to KERATINOCYTE GROWTH FACTOR PRECURSOR.	−1.12	−1.55	6.86E-04	3.12E-05
H3F3A	91945	Hs.546259	NM_002107	H3 histone, family 3A (H3F3A), mRNA.	−1.73	−2.31	3.64E-04	2.61E-05
LRRC59	32621	Hs.370927	NM_018509	Leucine rich repeat containing 59 (LRRC59), mRNA.	−0.96	−0.98	6.91E-04	2.12E-04
MAP3K7IP2	163253	Hs.269775	NM_145342;NM_015093	Mitogen-activated protein kinase kinase kinase 7 interacting protein 2 (MAP3K7IP2), mRNA.	−1.26	−1.34	1.52E-04	1.81E-04
RAB11FIP2	24644	Hs.173656	NM_014904	RAB11 family interacting protein 2 (class I) (RAB11FIP2), mRNA.	−1.37	−1.50	2.92E-04	8.41E-05
SDCBP	11180	Hs.200804	NM_001007068;NM_001007069;NM_001007070;NM_005625;NM_001007067	Syndecan binding protein (syntenin) (SDCBP), transcript variant 2, mRNA.	−0.99	−2.66	1.42E-03	3.26E-06
SGK3	20197	Hs.545401	NM_001033578;NM_170709;NM_013257	Serum/glucocorticoid regulated kinase family, member 3 (SGK3), transcript variant 1, mRNA.	−0.63	−0.59	1.20E-03	7.22E-04
TOMM20	143603	Hs.533192	NM_014765	Translocase of outer mitochondrial membrane 20 homolog (yeast) (TOMM20), mRNA.	−0.67	−0.86	3.50E-04	3.26E-05
TSGA14	34406	Hs.368315	NM_018718	Testis specific, 14 (TSGA14), mRNA.	−1.03	−1.29	2.08E-05	3.96E-06
TSHZ3	36819	Hs.278436	NM_020856	Teashirt zinc finger homeobox 3 (TSHZ3), mRNA.	−0.84	−0.92	1.62E-03	4.30E-04
TSPAN14	42822	Hs.310453	NM_030927	Tetraspanin 14 (TSPAN14), mRNA.	−1.04	−0.76	1.08E-05	6.68E-06
YWHAZ	98307	Hs.492407	NM_145690;NM_003406	Tyrosine 3-monooxygenase/tryptophan 5-monooxygenase activation protein, zeta polypeptide (YWHAZ), transcript variant 1, mRNA.	−0.68	−1.78	5.39E-05	3.88E-07

The list corresponds to the 15 down-regulated genes found after miR-155 overexpression at both 24 and 48 hours and predicted as miR-155 targets by 3 prediction tools (TargetScan, Pictar and MicroCible). RNG oligo IDs give access to transcripts and probes annotations through the microarray information system Mediante (http://www.microarray.fr:8080/merge/index), log2Ratio corresponds to the logarithm (base 2) of the ratio of miR-155/miR-Neg and Adj.P.Val is the false discovery rate p-values using the Benjamini-Hochberg correction.

### FGF7/KGF is a miR-155 target

**Figure 5:**
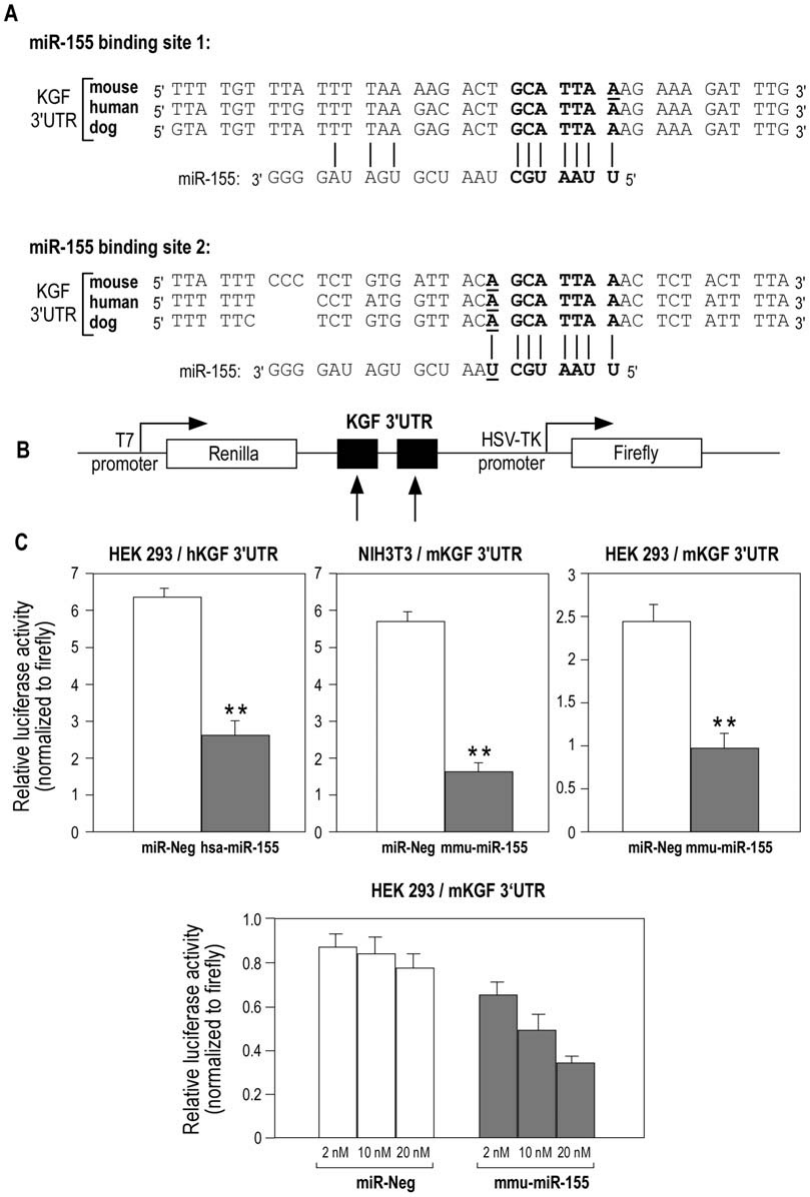
KGF mRNA is a primary target for miR-155. (A) Predicted interaction between miR-155 and the two putative binding sites in the KGF 3′UTR. Alignment of human, mouse and dog sequences in the 3′-UTR of KGF mRNA. The representation is limited to the region around the miR-155 complementary site. In bold, the “seed” region with a conserved anchoring adenosine (complementary to the first nucleotide of miR-155) and a conserved WC match to the eighth nucleotide of the miRNA. This latter is only present in binding site 2. (B) Schematic representation of the construct used in luciferase assay: a 919 bp and 787 bp region of the human and mouse KGF 3′-UTR containing the two putative miR-155 target sites (arrows) was cloned into the psiCHECK-2 vector. (C) Co-transfection of human or murine pre-miR-155 or pre-miR-Neg and of human or murine KGF 3′UTR-derived psiCHECK™-2 construct in HEK 293 and NIH 3T3 cells show a significant decrease in normalized luciferase activity 48 h post-transfection in both cell types. Lower panel: dose-dependent inhibition of normalized luciferase activity in HEK 293 cells. ** indicates a p-value of less than 0.01 in a Student's t test.

When we looked in the list of 15 transcripts whether one of these putative miR-155 targets was specific to the fibroblasts, FGF-7, a paracrine-acting epithelial mitogen produced by cells of mesenchymal origin, was identified. According to the TargetScanS algorithm, sequence alignment of the miR-155 complementary site in the 3′-UTR of KGF mRNA in human, mouse and dog allowed us to identify two conserved binding sites (Fig. 5A): i) binding site one (“seed” at position 1288–1293 and 1128–1133 after the transcription start site of the human and murine KGF mRNA respectively) which contains conserved “seed” and conserved anchoring adenosine ; ii) binding site two (“seed” at position 2017–2022 and 1824–1829 of the human and murine KGF mRNA respectively) which contains both conserved “seed” and conserved anchoring adenosine plus a conserved Watson-Crick match at the eighth nucleotide (Fig. 5A). To evaluate whether miR-155 can alter the expression of KGF, we cloned a fragment of 919 and 787 bp of the human and murine KGF 3′-UTR mRNA (NM_002009 and NM_008008) containing the two putative miRNA-binding sites into the psiCHEK**™**-2 vector (Fig. 3B) and transfected it into HEK 293 or NIH3T3 cells in the presence of either a human or murine synthetic pre-miR-155 analogue or a pre-miR-control. After normalization of the Renilla luciferase signal to the firefly luciferase signal, both human and murine pre-miR-155 induced a significant decrease in the normalized luciferase activity compared to control in the two cell types (Fig. 5B, upper panels). This effect was dose dependent as shown in Fig. 5B (lower panel).

### Only miR-155-binding site 2 is functional

**Figure 6:**
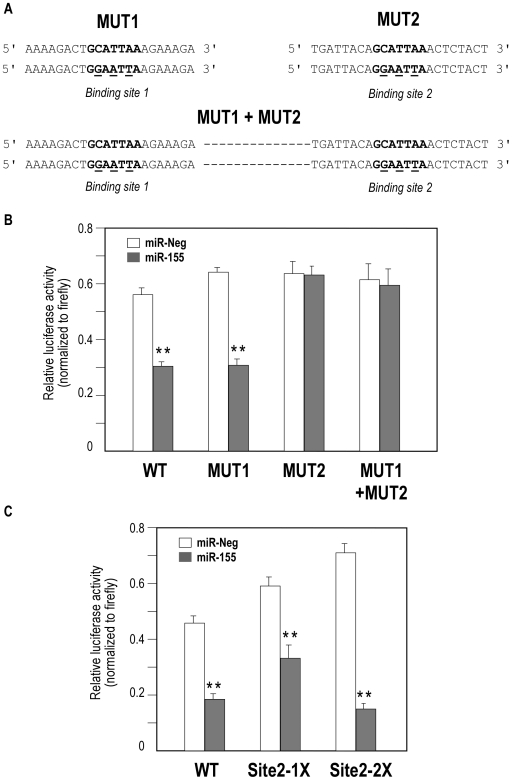
Only miR-155 putative binding site 2 is functional. (A) Generation by site-directed mutagenesis of murine fgf7 3′-UTR mutants in the “seed” of the putative binding sites (mutated bases are underlined): MUT 1 for the putative binding site 1, MUT 2 for the putative binding site 2 and a third mutant for both sites. (B) Co-transfection experiments of pre-miR-155 or pre-miR-Neg with these different constructs in HEK 293. Cells were harvested two days after transfection and luciferase activities were analyzed. All renilla luciferase activities were normalized with firefly luciferase activity. (C) HEK 293 cells were cotransfected with either pre-miR-155 or pre-miR-Neg together with different molecular constructs derived from pSI-check2 in which the second putative miR-155 binding site in FGF7 mRNA has been cloned once (1X) or twice (2X) behind the renilla luciferase gene. Cells were harvested two days after transfection, luciferase activities were analyzed and renilla luciferase activities were normalized with firefly luciferase activity. ** indicates a p-value of less than 0.01 in a Student's t test.

We then investigated whether one or both putative binding sites in KGF 3′UTR were functional. For this purpose, we generated two mutants targeting each seed of the 2 putative binding sites: MUT 1 for the putative binding site 1, MUT 2 for the putative binding site 2. A third mutant was built with both mutations: MUT1+MUT2 (Fig. 6A). Luciferase assay was performed with mutant and wild type 3′KGF UTR in HEK 293 cells. It demonstrated that miR-155 still efficiently inhibited luciferase activity after abrogation of the first seed while it had no inhibitory effect on the activity of MUT2 (Fig. 6B), indicating that only site 2 was functional. As expected, the luciferase activity of the double mutant was no more affected by miR-155. We next generated a minimal construct corresponding to binding site 2 (from position 1815 to 1837 in the murine KGF transcript, Site2-1X) and an additional construct containing a duplication of this sequence (Site2-2X). In agreement with previous data, miR-155 significantly inhibited the luciferase activity of the 2 constructs, the duplicated site construct being even more sensitive than the wild type 3′UTR KGF construct (Fig. 6C). Overall, these results demonstrated that only the second miR-155 binding site is functional in KGF 3′-UTR.

### MiR-155 specifically attenuates inflammatory cytokines-induced KGF release by lung fibroblasts

**Figure 7:**
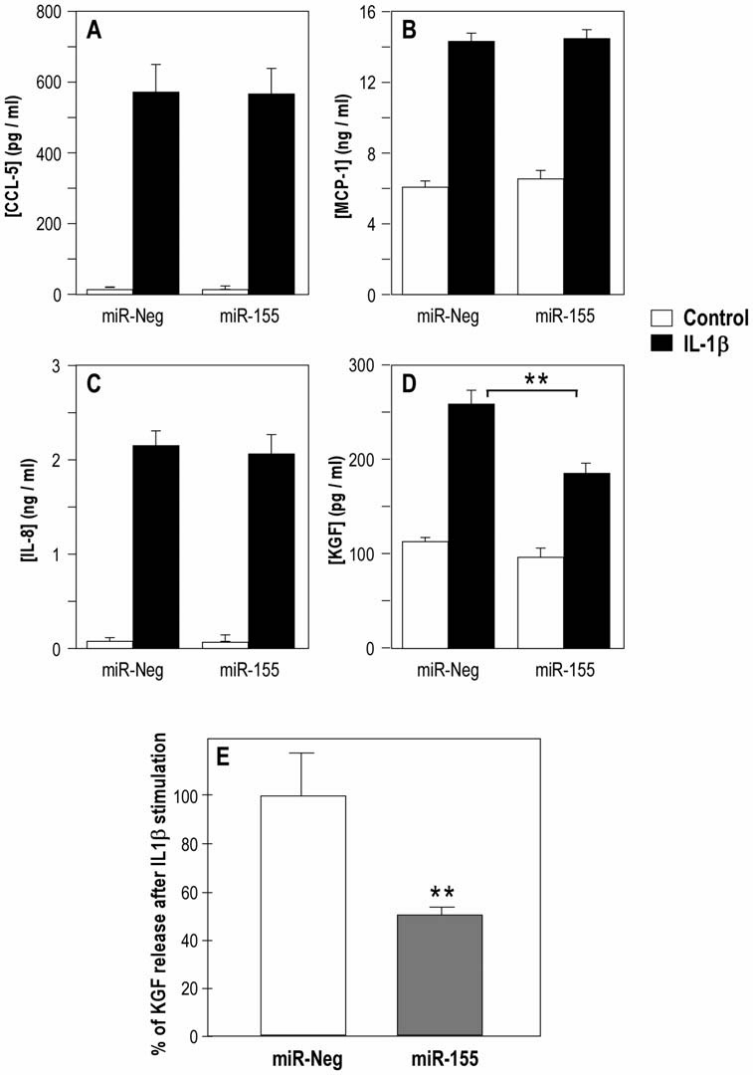
Transfection of human lung fibroblasts with pre-miR-155 inhibits IL-1β induced KGF release. Panels A-D show a representative experiment of pre-miR-Neg or pre-miR-155 transfection (10 nM) effect on CCL5/RANTES, MCP-1, IL-8 and KGF release by lung fibroblast stimulated with 10 ng/ml IL-1β. Results represent duplicates measurements of KGF by ELISA (R&D System) and duplicates measurements of CCL5, MCP-1 and IL-8 by FACS Array™ (human chemokine panel, Becton Dickinson) 48 h after transfection. Panel E summarizes the effect on KGF release by lung fibroblast stimulated by IL-1β after transfection of pre-miR-Neg or of pre-miR-155. Results are expressed in percentage of KGF released after IL-1β stimulation: pre-miR-155 decreases IL-1 β-mediated KGF release by approximately 45% compared to control. Results correspond to 3 independent experiments performed in duplicates. ** indicates a p-value of less than 0.01 in a Student's t test.

**Figure 8:**
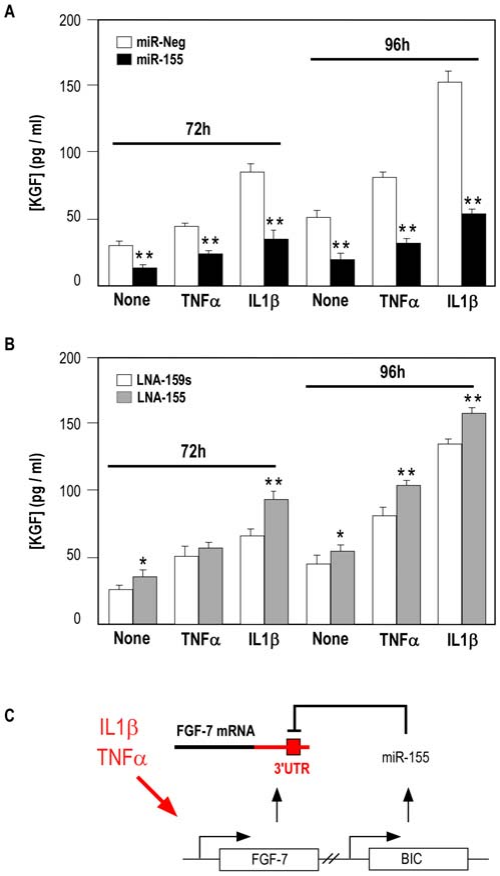
miR-155 overexpression or knock-down regulates KGF release in TNF-α- or IL-1β stimulated human lung fibroblasts. Panels A-B show a representative experiment of pre-miR-Neg/pre-miR-155 (10 nM) or LNA-159s/LNA-155 (25 nM) transfection effect on KGF release by lung fibroblast stimulated or not during 48 hours with 10 ng/ml TNF-α or IL-1β. Results correspond to triplicates measurements of KGF by ELISA (R&D System). *: p-value<0.05; **: p-value<0.01. Panel C: model of miR-155-dependent attenuation of KGF release following fibroblasts stimulation with inflammatory cytokines.

IL-1β is the most potent inducer of KGF in fibroblasts. Thus, we first assessed whether miR-155 overexpression in human fibroblasts could directly alter endogenous KGF release. Pre-miR-155 or a control pre-miRNA were transfected in lung fibroblasts in the presence or absence of IL-1β (10 ng/ml) and the production of KGF and of 3 inflammatory cytokines (CCL5/RANTES, MCP-1 and IL-8) was measured (Fig. 7). As expected, IL-1β stimulation led to a strong induction of all four factors and addition of pre-miR-155 did not affect the expression level of CCL5 (Fig. 7A), MCP1 (Fig. 7B) and IL-8 (Fig. 7C). Importantly, KGF release by lung fibroblasts in response to IL-1β was significantly decreased in cells transfected by miR-155 compared to control (Fig. 7D). The specific fraction of KGF release corresponding to IL-1 induction was indeed decreased to 45% of control (Fig. 7E). These results were confirmed by transfecting human lung fibroblasts either with a LNA-antimiR-155 in order to block the endogenous expression of miR-155 or with a pre-miR-155 in order to increase the expression of miR-155 (Fig. 8A and 8B). Transfected cells were treated for 72 or 96 hours with the inflammatory cytokines TNF-α- or IL-1β. Interestingly, miR-155 knock-down was able to potentiate KGF release in both TNF-α- and IL-1β-stimulated HFL1. As expected, transfection of HFL1 with a pre-miR-155 strongly affected KGF release in the 3 culture conditions (Fig. 8A). These results strongly suggest that miR-155 is involved in the attenuation of KGF expression in lung fibroblasts following inflammatory cytokines stimulation (Fig. 8C).

## Discussion

We have demonstrated for the first time that miR-155 could significantly decrease the relase of KGF induced by IL-1β or TNF-α in human normal pulmonary fibroblasts. Since KGF plays a central role in epithelial-mesenchymal interactions in lungs [47], our data suggest that an alteration in miR-155 expression level may have important pathophysiological impacts during acute lung injury or pulmonary diseases.

Several studies have reported that miR-155 expression is modulated in a number of physiological and pathological processes such as hematopoiesis, immunity, tumorigenesis and inflammation (for review, [48]). MiR-155 has been identified as a component of the primary response to several inflammatory mediators in different cell types [32]–[34]. It has been shown that miR-155 is induced by lipopolysaccharide (LPS), interferon (IFN), polyriboinosinic-polyribocytidylic acid (poly IC) or TNF-α in monocytes, macrophages and dendritic cells [32]–[34], [49]. In addition, nuclear factor-kappa B and AP-1 transcription factors have been shown to regulate miR-155 expression [32], [50], [51]. Our results showing the stimulation of miR-155 by IL-1β and TNF-α in human pulmonary fibroblasts fit well with these studies. These data are consistent with the high levels of miR-155 detected in synovial fibroblasts from Rheumatoid Arthritis patients, an autoimmune disorder associated with high inflammation at joint level [35]. Our observation showing an increased level of expression of miR-155 in a mouse model of lung fibrosis (Fig. 2) suggests a potential role for miR-155 during this physio-pathological process. The precise cellular location of miR-155 transcript has not been performed yet. It is tempting to speculate that the weak induction of KGF after stimulation by IL-1 observed in fibroblasts derived from patients with idiopathic pulmonary fibrosis (IPF) [52] is linked to an over-expression of miR-155 into these cells. Additional studies in the bleomycin-mouse model and on biopsies from IPF patients will help clarifying this important point.

Studies in hematopoietic, immune, and inflammatory cells as well as in hematologic and epithelial malignancies strongly suggest that miR-155 is an essential molecule in the control of myelopoeisis, erythropoiesis and B and T cell development. In particular, the use of BIC/miR-155 deficient mice or transgenic animals overexpressing miR-155 in B-cell lineage (E-miR-155) have provided the identification of several important miR-155 targets in different immune cell types linked with its function in the activation of T and B lymphocytes, macrophages and dendritic cells [28]–[30], [33], [37], [38]. Interestingly, BIC-deficient mice displayed significant remodeling of lung airways with age, associated with increased bronchiolar subepithelial collagen deposition and increased cell mass of sub-bronchiolar myofibroblasts. This is perfectly in line with our own observation, showing that miR-155 could have also additional functions in fibroblasts. It has been previously shown that miR-155 was expressed in primary lung fibroblasts in which it could regulate human angiotensin II type 1 receptor expression [40]. However, other putative functions of miR-155 in fibroblasts have not yet been documented. Our study provides a first attempt to identify the functional impact of miR-155 in normal human pulmonary fibroblasts.

In order to identify genes regulated by miRNAs in a specific cellular context, we have combined *in silico* and experimental approaches [53]. Several computational algorithms have been developed elsewhere to predict transcripts targeted by miRNAs [16], [17], [54], [55]. They generally postulate that the 5′ end of the miRNA, known as the “seed” region, forms perfect matches with the target sites and is usually conserved across species. However, slight differences in the different algorithms often lead to very distinct predictions. This clearly illustrates the limits of pure *in silico* approaches, and plainly justifies an experimental verification of these predictions. Incorporation of experimental data in the pipeline of analysis can be useful in order to take into account important parameters, such as the expression of the transcripts in a specific cellular context. Among several experimental approaches that are currently performed, the measurement of global gene expression after an ectopic expression of a specific miRNA deserves special interest. It usually induces a significant enrichment of predicted targets among the set of down-regulated transcripts. This suggests that most miRNAs, including miR-155 can indeed induce degradation of their mRNA targets [30], [44], [56]. We have validated this observation after alteration of several additional miRNAs (Lebrigand *et al*, manuscript in preparation). In our study, overexpression of miR-155 induces indeed a profound alteration of mRNA profiles associated with an over-representation of miRNAs predicted targets among the down-regulated transcripts. We calculated an enrichment score for miR-155 predicted targets in the set of downregulated transcripts, and compared it with the score of all other miRNAs. An ordered list of the miRNA compatible with a given set of downregulated transcripts was established, using “MicroTopTable”, a bioinformatics web tool available at Mediante [73]. MicroTopTable also provided the location of the binding sites on the transcripts and the energy of complexes. It works with several possible prediction tools. All three prediction tools used in the present study (i.e. TargetScan, Pictar and MicroCible) performed similarly in terms of fold enrichment and significance. The list of targets common to the three bioinformatics tools only represented 7% of the total number of transcripts predicted by at least one method. For the sake of clarity, we restricted our work to the group with the strongest typology, but other interesting candidates should be further analyzed later. We also noticed that miR-155-associated target enrichment already occurred at 24 hours (Fig. 2B), as already suggested by others [57], [58].

While the methodology described here worked finely for our project, its general application can possibly be weakened by a few limitations. A first possible drawback can be due to the high expression of miR-155 caused by the transfection, which does not reflect a physiological induction of this miRNA in HFL1. From that perspective, a supra-physiological concentration of miR-155 might induce adverse effects through alteration of “low affinity” targets. This is probably not the case here, since a final concentration of 0.15 nM of miR-155 induced a similar alteration of HFL1 transcriptome (data not shown). A second drawback could correspond to the saturation of the RISC complex by the heterologous expression of small RNAs, leading to an altered expression of genes controlled by endogenous miRNA, rather than by miR-155 [59]. This is also unlikely in these experiments, since we could not detect among upregulated transcripts any enrichment for a group of mRNA targeted by one of the miRNA highly expressed in fibroblasts. Based on our experience, we believe that the comparison of miRNAs and mRNAs expression profiles therefore provides a useful complement to pure computational predictions based solely on sequence information. It remains that even after such an analysis, the list of proposed miRNA targets still require a direct validation by Western blot analyses and/or a reporter plasmid assays.

Functional annotations of the microarrays experiments led to the point that miR-155 has an impact on several biological functions in fibroblasts related to pathways such as “cell-to-cell signalling and interaction”, “cell death” and “cellular movement” (Table S2). Of note, an induction of caspase-3 activity and an increase in cell migration on type-I collagen was observed in fibroblasts transfected with miR-155. Modifications in cell motility can probably be linked to targeting of genes such as RHEB and CYR61, two important regulators of cell adhesion, which were validated by luciferase assays (Figure S1A). One biologically relevant network drawn from the lists of genes that were significantly modulated at 24 and at 48 h after miR-155 transfection is shown in Figure S1B. It contains five putative miR-155 targets. One can notice the strong modulation of genes associated with cell survival, such as Akt, and with migration, such as proteases and integrins (in particular ITGB1, the integrin β1, one of the type I collagen receptor subunit). In line with this observation, a targeting by miR-155 of RhoA, a Rho GTPase, has recently been reported elsewhere [39]. Importantly, our list of putative miR-155 targets (Table S3) contains transcripts that were also identified in murine hematopoietic systems (namely BACH1, H3F3A, HIF1A, MAP3K7IP2, MYO10, RAB11FIP2, SDCBP, SGK3, TOMM20, TSPAN14, WEE1, YWHAZ) [29], [30], [59], [60]. Such a conservation among species is certainly indicative of the likely impact of such interactions. Our list also share some common candidates with a study that identified several miR-155 targets in human mature dendritic cells [34]: BACH1, CMAT1, HNRPA3, MAP3K7IP7, PAPOLA and SERTAD2.

A central aspect of our work is related to the experimental screening of miR-155 targets specific to fibroblasts. This allowed the identification of KGF/FGF7, a central mediator of epithelial-mesenchymal interaction as a direct target of miR-155. KGF is a paracrine-acting, epithelial mitogen produced by cells of mesenchymal origin. KGF acts exclusively through FGFR2b, a subset of FGF receptor isoforms (for review [47]). KGF has been shown to be upregulated after epithelial injury in numerous conditions. Preclinical data from several animal models demonstrated that KGF could enhance the regenerative capacity of epithelial tissue [61]–[64]. Despite the important function of KGF in lung, the regulation of its production by lung fibroblasts is poorly understood [52]. Inflammatory mediators are known stimulants of KGF in fibroblasts. Among these mediators, IL-1β appears to be the most potent inducer of KGF expression in fibroblasts derived from multiple tissues [65], [66]. Previous studies have reported the presence of AP-1 sites in the promoter region of mouse and human KGF genes [67]. In lung or dermal fibroblast, it has been demonstrated that the IL-1β induced KGF secretion is controlled through a balance between c-Jun and JunB. Indeed, KGF expression was enhanced in JunB −/− fibroblast whereas KGF was hardly detectable in c-jun −/− fibroblasts [68].

Our observation of a specific role for miR-155 in that context appears of particular interest. Indeed, using KGF 3′-UTR luciferase constructs, we have demonstrated that KGF was a direct target of miR-155. Interestingly, luciferase assay performed with different KGF 3′-UTR mutants for the two putative miR-155 binding sites showed that only binding site two was responsible of miR-155-mediated KGF regulation. The binding site one contains the conserved “seed” and the anchoring adenosine whereas the binding site two contains also a conserved Watson-Crick match at the eighth nucleotide (m8M, according to the nomenclature established by [16]). This result indicates that the predicted binding site one is a false positive due to the lack of a conserved Watson-Crick match at the eighth nucleotide of the miRNA and is in total agreement with previous studies reporting that the presence of m8M decreased the possibility of false positive compared to the sole presence of the “seed” and of the anchoring adenosine [16], [53]. This point seems of particular importance for sites containing little 3′ pairing support and “3′ compensatory” sites such as the 2 binding sites discussed here.

We also demonstrated that miR-155 over-expression efficiently inhibited endogenous KGF following stimulation with inflammatory cytokines IL-1β and TNF-α. Conversely, miR-155 knock-down using LNA-antimiR-155 was able to potentialize KGF release in both TNF-α- or IL-1β-stimulated HFL1 at both times (Fig. 5–6). Therefore, we propose a model in which both KGF mRNA and miR-155-derived primary transcript BIC are induced following inflammatory cytokines. According to this model, a subsequent accumulation of miR-155 would attenuate KGF production in a negative feedback mechanism (Fig. 8C). An alternative model would be that miR-155 attenuates the inflammatory intracellular pathway, as demonstrated recently [34] by directly targeting adaptor molecules of the TLR/IL1 signaling cascade such as MAP3K7IP7/TAB2. However, although TAB2 expression was found to be inhibited by miR-155 in our microarray experiments, we clearly showed that miR-155 specifically inhibited IL1-induced KGF production but did not affect the expression level of three other cytokines (CCL5, MCP1 and IL-8), indicating that in our model, miR-155 does not affect the TLR/IL1 signalling cascade.

In conclusion, this study provides a global characterization of miR-155 targets in human lung fibroblasts. It leads to the identification of KGF/FGF7 as a mesenchymal-specific miR-155 target. To date, this is the first report relating evidence for miRNA involvement in lung epithelial-mesenchymal interactions. Finally, this work provides new insights into the pathogenesis of lung fibrosis, by suggesting a role for miRNAs in the lung fibrotic process.

## Materials and Methods

### Ethics statement

All animal care and experimental protocols were conducted in accordance with the regulations of the institutional care and use committee at the University of Lille 2. Personnel from the laboratory carried out all experimental protocols under strict guidelines to insure careful and consistent handling of the mice.

### Cell Culture and Treatments

The human fibroblast cells HFL1 (CCL-153) derived from normal lung tissue [69], adenocarcinoma A549 cells, NIH 3T3 and HEK 293 were purchased from the American Type Culture Collection (Manassas, VA) and maintained in monolayer culture in Dulbecco's modified Eagle's medium (DMEM) supplemented with 10% fetal calf serum (FCS). IL-1β, TNF-α and TGF-β (Upstate) were used at final concentration of 10 ng/ml.

### RNA isolation from cell samples

Total RNA were extracted from the samples with TRIzol solution (Invitrogen, Carlsbad, CA, USA), and the integrity of RNA was assessed by using an Agilent BioAnalyser 2100 (Agilent Palo Alto, CA) (RIN above 7). The miRvana miRNA isolation kit was used for isolation and enrichment of small RNA fractions (Ambion, Austin, TX), according to the manufacturer's protocol.

### Animal treatment

9–12 weeks old male C57BL/6 and Balb/C mice were purchased from Charles River, France. To induce fibrotic changes, mice were intratracheally instillated with bleomycin or PBS as previously described [42]. Briefly, mice were anesthetized with sevoflurane inhalation (Abbott, UK) and placed in dorsal recumbency. Transtracheal insertion of a 24-G animal feeding needle was used to instillate bleomycin (0.75 unit/ml) or vehicle (PBS), in a volume of 80 µl. Mice were sacrificed 7 and 14 days after instillation and lungs were removed for further analysis. Lung fibrosis was assessed by histopathology after fixation with 4% paraformaldehyde/PBS overnight at 4°C. The major criteria examined included interstitial thickening of alveolar or bronchiolar walls, collagen deposition, and inflammatory cell infiltration. Total RNA was extracted from lungs removed from PBS- and bleomycin-treated mice (n = 4 for both strains) as described above and used for assessment of miR-155 expression by quantitative RT-PCR.

### Microarray procedure

#### miRNA microarray procedure

The oligonucleotide sequences corresponding to 2054 mature miRNAs (409 homo sapiens) found in the miRNA registry (Release 8.2; [10]) are available on http://www.microarray.fr:8080/merge/index (follow the link to “Ipmc MicroRNA-1k microarray – v4)”. Each oligonucleotide was spotted four times on each microarray (2 distinct pairs of spots), in order to reduce positional bias of the fluorescence readout. Experimental data and associated microarray designs have been deposited in the NBCI Gene Expression Omnibus (GEO) (http://www.ncbi.nlm.nih.gov/geo/) under series GSE12033/GSE12035 and platform record GPL4717. Target preparation and array hybridization were performed as previously described [70], [71]. Briefly, small RNA fraction was purified from 100 µg total RNA with the miRvana miRNA isolation kit (Ambion, Austin, TX) and 5 µg of small RNA fraction were then chemically labeled with the Alexa fluors (Ulysis kit, Invitrogen) as previously described [72]. Labeled RNA were then combined, purified and hybridized in 300 µl hybridization buffer (Agilent hybridization buffer) for 16 hrs at 48°C in a dye-swap experiment. Arrays were then washed once at room temp in Agilent wash buffer (Agilent) for 5 min and then washed in Agilent wash buffer II at 37°C for 30 sec, spun dry and scanned with a Genepix scanner (Axon Instruments, Molecular Devices Corporation 3280 Whipple Road Union City, CA 94587 USA). TIF images containing the data from each fluorescence channel were quantified with the Genepix pro 6.0 program (Axon Instruments) using a ‘circular features’ quantification method.

#### Expression microarray procedure

Pangenomic microarrays were printed using human RNG/MRC oligonucleotide collection as previously described [43]. RNA were labelled and hybridized as described in Moreilhon et al [73]. Two biological replicates were performed for each comparison. Experimental data and associated microarray designs have been deposited in the NCBI Gene Expression Omnibus (GEO) (http://www.ncbi.nlm.nih.gov/geo/) under serie GSE14477 and platform record GPL1456.

#### Statistical analysis

Normalizations were performed using the limma package available from Bioconductor (http://www.bioconductor.org). Intra slide and inter slide normalization were performed using the Print Tip Loess and the quantile methods respectively. Means of ratios from all comparisons were calculated and and B test analysis was performed using the Limma package available from Bioconductor. Differentially expressed genes were selected using a Benjamini-Hochberg correction of the p-value for multiple tests, based on a p-value below 0.05 and a fold change cut off (logRatio>0.5).

#### Biological Theme Analysis

Data from expression microarrays were analyzed for enrichment in biological themes (Gene Ontology molecular function and biological process) and build biological networks using Ingenuity Pathway Analysis software (http://www.ingenuity.com/) and Mediante [74], an information system containing diverse information about our probes set and the data sets (http://www.microarray.fr:8080/merge/index). Over-represented biological themes are shown in supplementary Table S1.

#### MiR-155 targets analysis

Two in-house bioinformatics tools have been developed to predict miRNA targets (http://www.microarray.fr:8080/merge/index follow the link to microRNA and Bioinformatic tools): i) MicroCible is a miRNA target predictor that scans transcripts sequences for the presence of “miRNA seed” complementary sequence. This search can be performed for different “seed” match type [53], a minimal free energy binding cutoff, and the location of the potential targeting site (i.e. 3′UTR or entire transcript) ; ii) MicroTopTable is dedicated to the search of putative enrichments in miRNA predicted binding sites among a set of modulated genes for different prediction softwares (i.e. Miranda, MicroCible, TargetScan or PicTar). MicroTopTable ranks the transcripts into three categories (“Upregulated”, “Down regulated” and “non-modulated”), according to thresholds for expression level and for differential expression. MicroTopTable then calculates the number of predicted targets for each miRNA, according to the prediction software selected, in each of the three categories. Enrichment in miRNA targets in each category is then tested using the hypergeometric function.

### Quantitative RT-PCR of mature miRNA

MiR-155 expression in cytokines-stimulated fibroblasts was evaluated using TaqMan MicroRNA Assay (Applied Biosystems, Foster City, CA) as specified in their protocol. Real-time PCR was performed using GeneAmp Fast PCR Master Mix (Applied Biosystems, Foster City, CA) and ABI 7900HT real-time PCR machine. All reactions were performed in duplicate. Expression levels of mature microRNAs were evaluated using comparative CT method (2-deltaCT). Transcript levels of let-7a and RNU6B was used as endogenous control.

### Molecular constructs

Various molecular constructs were derived from psiCHECK**™**-2 (Promega) by cloning behind the renilla luciferase ORF sequences from fgf7 3′-UTR mRNA. For human and murine full lenght KGF 3′UTR cloning, a 919 and 787 nt sequence encompassing two putative miR-155 binding sites from human and mouse FGF7 3′ UTR respectively was amplified using the primers listed in “Online Supplementary Materials S1” and cloned in the XhoI and NotI restrictions sites. Mutations were introduced by site-directed mutagenesis in mice fgf7 3′UTR miR-155 putative binding sites using the QuickChange Kit (Stratagene). Primers used for site-directed mutagenesis are listed in “Online Supplementary Materials S1”. In order to analyze in details the sequence targeted by miR-155 in fgf7 3′UTR we derived several constructs from the second miR-155 binding site. Complementary oligonucleotides referenced in “Online Supplementary Materials S1” (50 µM final concentrations) were mixed with 10X Oligo Annealing Buffer (Invitrogen) heated 95°C for 4 minutes and allowed to cool at room temperature for 10 min. Diluted (10 nM) dsDNA were subsequently cloned in XhoI/NotI restriction sites in the pSI-Check2 (Promega). Site2-1X corresponds to the sequence 1815 to1837 of the murine fgf7 messenger. In the Site2-2X, the 22 nt sequence emcompassing the miR-155 “seed” match has been duplicated.

### Transfection and luciferase assays

#### Pre-miRNAs overexpression in HFL1

pre-miR-155 and control miRNA (miR-Neg # 1) were purchased from Ambion. HFL1 cells were grown in 10% FCS in DMEM and transfected at 50% confluency in 6-well plates using Lipofectamin RNAi MAX**™**(InVitrogen) with pre-miRNA at a final concentration of 10 nM.

#### Pre-miRNAs and psiCHECK™-2 plasmid constructs co-transfection

NIH 3T3 and HEK 293 cells were cultured in 10% FCS in DMEM until confluency. Then, cells were plated into 48-well plates at a density of 26.5 10^3^ cells/well and cotransfected using lipofectamin 2000**™** (InVitrogen) with 0.4 µg of psiCHECK**™**-2 plasmid construct and pre-miR-155 or control miRNA at a final concentration of 10 nM. 48 hours after transfection, Firefly and Renilla Luciferase activities were measured using the Dual-Glo**™** Luciferase assay (Promega).

#### Assessment of KGF production from lung fibroblast

Pre-miR-155 and control miRNA (miR-Neg # 1) were purchased from Ambion; LNA anti-miR-155 and a control LNA inhibitor (LNA-159s) were purchased from Exiqon. Adult or embryonnal lung fibroblast cells were cultured in 10% FCS in DMEM until confluency. Then, cells were plated into 6-well plates at a density of 4.10^6^ cells/well and transfected using lipofectamin RNAi MAX**™** (InVitrogen) with pre-miRNAs or LNA-anti-miRs at a final concentration of 10 nM and 25 nM respectively. Twenty four hours after transfection, cells were washed in PBS, and medium was replaced by DMEM without serum. Then cells were stimulated with IL-1β or TNF-β at a final concentration of 10 ng/ml. At different times after stimulation, supernatants were collected and KGF was measured by ELISA kit purchased from R&D Systems according to manufacturer's protocol.

### Cytokine assay

MCP-1, IL-8 and CCL5 assays were performed using the BD FACSArray**™** Bioanalyser (BD Biosciences, Le Pont de Claix, France) according to manufacturer's protocol. Cytokine assays were measured in 50 µl of conditioned media.

### Caspase 3/7 assay

The activation of executioner caspase-3 and -7 was determined using the Caspase-Glo 3/7 Assay kit according to manufacturer's instructions from Promega (Madison, WI). Cells were plated in triplicate in 96-well plates and transfected as described above. Samples were read after 1 hr of incubation with the caspase substrate on a luminometer .

### *In Vitro* Wound-Scratching Assay

Cells were plated on noncoated or collagen-type I-coated 12-well plates and transfected as described above. Twenty four hours after transfection, confluent cells were wounded by a pipet tip. The *in vitro* wound-healing process was then recorded by videomicroscopy for 24 h from the scratching on an Axiovert 200 M inverted microscope (Carl Zeiss, Le Pecq, France) equipped with 37°C and 5% CO2 regulated insert (Pecon GmbH, Germany). Brightfied images were taken each hour through a 10× phase contrast objective with a CoolSNAPHQ CCD Camera managed by Metamorph Software (Roper Scientific, Evry, France). The motility of the cells was evaluated by the determination of the repaired area percentage and of the cell migration speed using the ImageJ sotware.

### Statistical analysis

Results are given as mean±S.E.M. Statistical analyses were performed by using Student's t-test as provided by Microsoft Excel™ and the null hypothesis was rejected at the 0.05 level.

## Supporting Information

Online Supplemental Material S1 (0.04 MB DOC)

Figure S1 Validation of miR-155 targets and hypothetic model of miR-155 in the regulation of fibroblasts apoptosis and motility. (A) miR-155 targets 3′UTR mRNAs of RHEB, POLE3, CYR61 and H3F3A. HEK 293 cells were co-transfected with pre-miR-155 or pre-miR-Neg and different pSi-CHECK constructs as described in the Materials and Methods section. Transfection of pre-miR-155 induces a significant decrease in normalized luciferase activity 48 h post-transfection for all 3′UTR tested. (B) Ingenuity Pathway Analysis identifies a network of genes potentially modulated by miR-155 and involved in cell survival and migration. The network is displayed graphically as nodes (genes/gene products) and edges (the biological relationships between the nodes). Red and green nodes correspond to up- and down-regulated genes after a 24 (left) or 48 h (right) pre-miR-155 transfection experiment. As described in the legend provided, nodes are displayed using various shapes that represent the functional class of the gene product. Edges are displayed with various labels that describe the nature of the relationship between the nodes (A, activation; B, binding; E, expression; I, inhibition; P, phosphorylation; T, transcription). Edges without a label represent binding only. Grey nodes were identified by the pathway analysis as part of the network. The putative inhibitory action of miR-155 on 5 gene products has been represented. AREG: amphiregulin; ADAM: ADAM metallopeptidase domain; CTGF: connective tissue growth factor; CYR61: cysteine-rich, angiogenic inducer, 61; FERMT2: fermitin family homolog 2; IGFBP3: insulin-like growth factor binding protein 3; ITGAV: integrin, alpha V; ITGB: integrin, beta; JAM: junctional adhesion molecule; MMP1: matrix metallopeptidase 1; MYO10: myosin X; PKN2: protein kinase N2; PRKCI: protein kinase C, iota; RHEB: Ras homolog enriched in brain; SH3D19: SH3 domain containing 19; SULF1: sulfatase 1; THBS2: thrombospondin 2; VCAN: versican.
(0.98 MB TIF)

Table S1 Main differentially expressed miRNAs between HFL1 and A549 cells. RNG oligo IDs give access to transcripts and probes annotations through our system of information Mediante (http://www.microarray.fr:8080/merge/index). Expression values correspond to the mean of fluorescence intensity for each probe.
(0.05 MB PDF)

Table S2 GO Database functional analysis of the genes regulated in response to miR-155 overexpression. Most significant themes identified by Ingenuity Pathway analysis. The genes modulated between miR-155 and miR-Neg transfected samples at two time points (24 h and 48 h) are listed in each case. The p value was calculated as a Fisher's exact probability.
(0.10 MB PDF)

Table S3 Full list of the miR-155 predicted targets down-regulated following miR-155 overexpression in HFL1. The 260 transcripts predicted to be miR-155 targets by at least one of the following algorithm: TargetScan, Pictar and MicroCible, are listed. Logarithm (base 2) of the ratio of miR-155/miR-Neg and false discovery rate p-values using the Benjamini-Hochberg correction are represented. ID: correspond to RNG oligo IDs that give access to transcripts and probes annotations through our system of information Mediante (http://www.microarray.fr:8080/merge/index).
(0.10 MB PDF)
