# Supplementary material for: Identification of Keratinocyte Growth Factor as a Target of microRNA-155 in Lung Fibroblasts: Implication in Epithelial-Mesenchymal Interactions
Source: PLoS One. 2009 Aug 24;4(8):e6718. doi: 10.1371/journal.pone.0006718 (PMC2726943; doi:10.1371/journal.pone.0006718)
Supplement: Online Supplemental Material S1 — (0.04 MB DOC) [file pone.0006718.s001.doc]

**Online Supplemental Material**

Set of primers used in this study:

***Cloning 3’UTR human FGF7 in psiCHECK:***

Primers used for KGF 3’UTR cloning Sequence (5’3’)

Sense : CTCCAGTGATCAAGCTGGACTTGTGC

Antisense : GCGGCCGCCCGTTGAGCCAGACAGTTTT

***Cloning 3’UTR murine fgf7 in psiCHECK:***

Primers used for KGF 3’UTR cloning Sequence (5’3’)

Sense : ACTACTCGAGCTGATCAAGCTGGACTTGCGC

Antisense : ATAAGAATGCGGCCGCTAACAAAACAATAAAATTCAAATACAAC

***KGF (site2):***

Forward : tcgatgattaca**GCATTA**aactctacctcgagataagaatgc

Reverse : tcgatgattaca**GCATTA**aactctacctcgagataagaatgc

2_KGF

Sense: tcgatgattacaGCATTAaactctacctcgagtgattacaGCATTAaactctacgc

Reverse: ggccgcgtagagtttaatgctgtaatcactcgaggtagagtttaatgctgtaatca

The seed-match sequences for miR-155 are highlighted in bold.

***Primers used for site-directed mutagenesis***

MiR155/Site 1:

Forward : GCATTTTTGTTTATTTTAAAAGACTG**G**A**A**T**T**AAGAAAGATTTGAAGAATATACAC

Reverse:

GTGTATATTCTTCAAATCTTTCTT**A**A**T**T**C**CAGTCTTTTAAAATAAACAAAAATGC

MiR155/Site 2 :

Forward :

GCTTATTTCCCTCTGTGATTACAG**G**A**A**T**T**AACTCTACTTTAAGTTGTATTTG

Reverse:

CAAATACAACTTAAAGTAGAGTT**A**A**T**T**C**CTGTAATCACAGAGGGAAATAAGC

***Cloning 3’UTR human RHEB in psiCHECK:***

Primers used for RHEB 3’UTR cloning Sequence (5’3’)

Sense : CTCGAGGCCTGAGGACACTGGGAATA

Antisense : GCGGCCGCCCTTTACAGCTGCTCCTTGG

***Cloning 3’UTR human POLE3 in psiCHECK:***

Primers used for POLE3 3’UTR cloning Sequence (5’3’)

Sense : CTCGAGTGTGAAGCTTTTCCATGCTG

Antisense : GCGGCCGCCAGAGGAGAGGCAGGAGAGA

***Cloning 3’UTR human CYR61 in psiCHECK:***

Primers used for CYR61 3’UTR cloning Sequence (5’3’)

Sense : CTCGAGTTTCCAGGGCACACCTAGAC

Antisense : GCGGCCGCCCCCAAAGGCTACATTTTGA

***Cloning 3’UTR human H3F3A in psiCHECK:***

Primers used for H3F3A 3’UTR cloning Sequence (5’3’)

Sense : CTCGAGTTCCATGGGGTCAAAAGGTA

Antisense : GCGGCCGCGCAAACTTACAGGAACAGCACA

**Legends to:**

**Table S1: Main differentially expressed miRNAs between HFL1 and A549 cells.** RNG oligo IDs give access to transcripts and probes annotations through our system of information Mediante (http://www.microarray.fr:8080/merge/index). Expression values correspond to the mean of fluorescence intensity for each probe.

**Table S2: GO Database functional analysis of the genes regulated in response to miR-155 overexpression in HFL1.** List of themes identified by Ingenuity Pathway Analysis 24 and 48 h after pre-miR-155 transfection. The genes modulated in each theme are represented. The probability to obtain the number of genes in a certain pathway in the list of differentially expressed genes was compared with the representation of the same pathway among all the genes on the microarray and was calculated as a Fisher’s exact probability.

**Table S3: Full list of the miR-155 predicted targets down-regulated following miR-155 overexpression in HFL1.** The 260 transcripts predicted to be miR-155 targets by at least one of the following algorithm: TargetScan, Pictar and MicroCible, are listed. Logarithm (base 2) of the ratio of miR-155 / miR-Neg and false discovery rate p-values using the Benjamini-Hochberg correction are represented. ID: correspond to RNG oligo IDs that give access to transcripts and probes annotations through our system of information Mediante (http://www.microarray.fr:8080/merge/index).

**Figure S1: Validation of miR-155 targets and hypothetic model of miR-155 in the regulation of fibroblasts apoptosis and motility. (A)** miR-155 targets 3’UTR mRNAs of RHEB, POLE3, CYR61 and H3F3A. HEK 293 cells were co-transfected with pre-miR-155 or pre-miR-Neg and different pSi-CHECK constructs as described in the Materials and Methods section. Transfection of pre-miR-155 induces a significant decrease in normalized luciferase activity 48h post-transfection for all 3’UTR tested. (B) Ingenuity Pathway Analysis identifies a network of genes potentially modulated by miR-155 and involved in cell survival and migration. The network is displayed graphically as nodes (genes/gene products) and edges (the biological relationships between the nodes). Red and green nodes correspond to up- and down-regulated genes after a 24 (left) or 48h (right) pre-miR-155 transfection experiment. As described in the legend provided, nodes are displayed using various shapes that represent the functional class of the gene product. Edges are displayed with various labels that describe the nature of the relationship between the nodes (A, activation; B, binding; E, expression; I, inhibition; P, phosphorylation; T, transcription). Edges without a label represent binding only. Grey nodes were identified by the pathway analysis as part of the network. The putative inhibitory action of miR-155 on 5 gene products has been represented. AREG: amphiregulin ; ADAM: ADAM metallopeptidase domain ; CTGF: connective tissue growth factor ; CYR61: cysteine-rich, angiogenic inducer, 61; FERMT2: fermitin family homolog 2 ; IGFBP3: insulin-like growth factor binding protein 3 ; ITGAV: integrin, alpha V ; ITGB: integrin, beta ; JAM: junctional adhesion molecule ; MMP1: matrix metallopeptidase 1 ; MYO10: myosin X ; PKN2: protein kinase N2 ; PRKCI: protein kinase C, iota ; RHEB: Ras homolog enriched in brain ; SH3D19: SH3 domain containing 19 ; SULF1: sulfatase 1 ; THBS2: thrombospondin 2 ; VCAN: versican.
