# Supplementary figures and images for: Identification of Keratinocyte Growth Factor as a Target of microRNA-155 in Lung Fibroblasts: Implication in Epithelial-Mesenchymal Interactions
Source: PLoS One. 2009 Aug 24;4(8):e6718. doi: 10.1371/journal.pone.0006718 (PMC2726943; doi:10.1371/journal.pone.0006718)

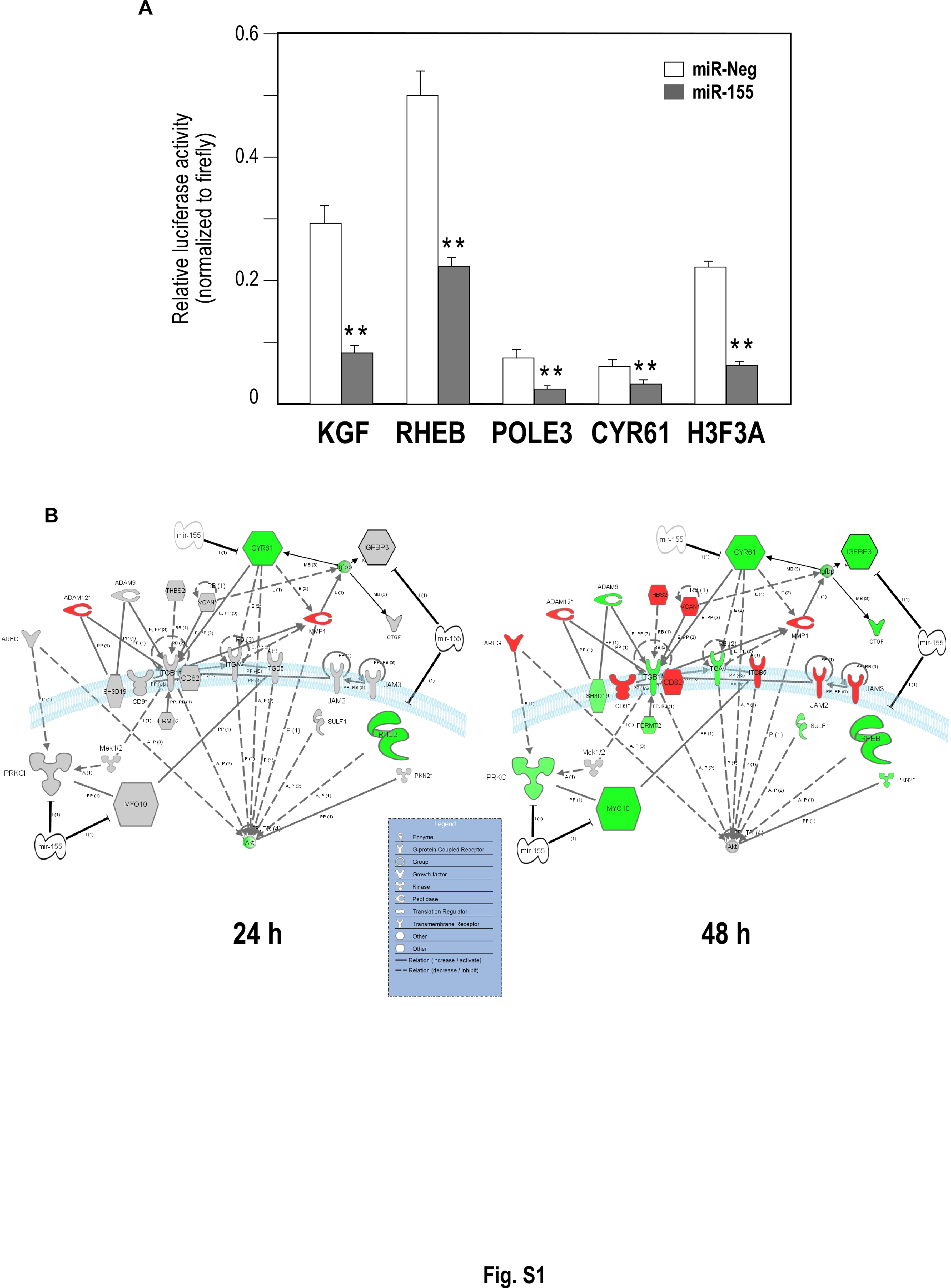

Supplement: Figure S1 — Validation of miR-155 targets and hypothetic model of miR-155 in the regulation of fibroblasts apoptosis and motility. (A) miR-155 targets 3′UTR mRNAs of RHEB, POLE3, CYR61 and H3F3A. HEK 293 cells were co-transfected with pre-miR-155 or pre-miR-Neg and different pSi-CHECK constructs as described in the Materials and Methods section. Transfection of pre-miR-155 induces a significant decrease in normalized luciferase activity 48 h post-transfection for all 3′UTR tested. (B) Ingenuity Pathway Analysis identifies a network of genes potentially modulated by miR-155 and involved in cell survival and migration. The network is displayed graphically as nodes (genes/gene products) and edges (the biological relationships between the nodes). Red and green nodes correspond to up- and down-regulated genes after a 24 (left) or 48 h (right) pre-miR-155 transfection experiment. As described in the legend provided, nodes are displayed using various shapes that represent the functional class of the gene product. Edges are displayed with various labels that describe the nature of the relationship between the nodes (A, activation; B, binding; E, expression; I, inhibition; P, phosphorylation; T, transcription). Edges without a label represent binding only. Grey nodes were identified by the pathway analysis as part of the network. The putative inhibitory action of miR-155 on 5 gene products has been represented. AREG: amphiregulin; ADAM: ADAM metallopeptidase domain; CTGF: connective tissue growth factor; CYR61: cysteine-rich, angiogenic inducer, 61; FERMT2: fermitin family homolog 2; IGFBP3: insulin-like growth factor binding protein 3; ITGAV: integrin, alpha V; ITGB: integrin, beta; JAM: junctional adhesion molecule; MMP1: matrix metallopeptidase 1; MYO10: myosin X; PKN2: protein kinase N2; PRKCI: protein kinase C, iota; RHEB: Ras homolog enriched in brain; SH3D19: SH3 domain containing 19; SULF1: sulfatase 1; THBS2: thrombospondin 2; VCAN: versican. (0.98 MB TIF) [file pone.0006718.s002.tif]
