# Supplementary material for: Identification of Keratinocyte Growth Factor as a Target of microRNA-155 in Lung Fibroblasts: Implication in Epithelial-Mesenchymal Interactions
Source: PLoS One. 2009 Aug 24;4(8):e6718. doi: 10.1371/journal.pone.0006718 (PMC2726943; doi:10.1371/journal.pone.0006718)
Supplement: Table S1 — Main differentially expressed miRNAs between HFL1 and A549 cells. RNG oligo IDs give access to transcripts and probes annotations through our system of information Mediante (http://www.microarray.fr:8080/merge/index). Expression values correspond to the mean of fluorescence intensity for each probe. (0.05 MB PDF) [file pone.0006718.s003.pdf]

ID	Name	Expression value	
		HFL1	A549
8311	hsa-let-7i	1291	4047
7293	hsa-mir-125b-1	5990	1592
7301	hsa-mir-129-1	1208	440
8296	hsa-mir-130a	1484	675
7331	hsa-mir-143	1557	192
7333	hsa-mir-145	1881	189
7351	hsa-mir-155	1907	235
7456	hsa-mir-192	202	1125
8242	hsa-mir-193b	398	1224
7459	hsa-mir-194-1	193	1123
7468	hsa-mir-199a-2	3697	163
7496	hsa-mir-21	1175	5648
7506	hsa-mir-214	1043	249
7519	hsa-mir-221	10042	1761
7521	hsa-mir-222	7734	2000
8202	hsa-mir-27b	670	3006
8187	hsa-miR-30a-5p	500	1867
7650	hsa-mir-30d	591	1766
7677	hsa-mir-31	1142	5617
8094	hsa-mir-513-2	1752	285
14233	hsa-mir-595	3235	819
