# Supplementary material for: Identification of Keratinocyte Growth Factor as a Target of microRNA-155 in Lung Fibroblasts: Implication in Epithelial-Mesenchymal Interactions
Source: PLoS One. 2009 Aug 24;4(8):e6718. doi: 10.1371/journal.pone.0006718 (PMC2726943; doi:10.1371/journal.pone.0006718)
Supplement: Table S2 — GO Database functional analysis of the genes regulated in response to miR-155 overexpression. Most significant themes identified by Ingenuity Pathway analysis. The genes modulated between miR-155 and miR-Neg transfected samples at two time points (24 h and 48 h) are listed in each case. The p value was calculated as a Fisher's exact probability. (0.10 MB PDF) [file pone.0006718.s004.pdf]

Category	P-value 24h	P-value 48h	Molecules (24h)	Molecules (48h)
Amino Acid Metabolism	6.32E-03	4.82E-04	ANXA2, DHFR, DIO2, GFPT2	GLS, GFPT2
Carbohydrate Metabolism	6.32E-03	ns	NT5E, UAP1	
Cell Cycle	4.76E-04	3.39E-08	ADM, AKAP12, YWHAG, BTG3, WEE1, CDK6, IRF9, ID3, IVNS1ABP, HMOX1, SPRY2, GMNN, CXCL12, UBE2V1, ZAK, IFITM1, NEUROG1, CYR61, IL11	KIF23, DLGAP5, PLK3, NCAPG, SMAD3, UBE2V2, CCNB2, KRAS, ROCK2, CCNG1, CAV1, ITGAV, TUBB3, YWHAG, THBS1, WEE1, CDK6, IRF9, ANLN, EIF3E, STAT3, TPX2, AREG, PPP2CB, IRF7, PTPN11, BHLHB2, ZAK, IGFBP3, CYR61, ADM, AKAP12, SULF1, TPM1, SH3BP4, ID2, BTG3, TPD52L1, HIF1A, NGF, EIF4E, ID1, CCNA2, HMOX1, GFRA1, GMNN, SIPA1, UBE2V1, TOP2A, IFITM1, UBE2D3, CTNNB1, STAT1, FGF7, CITED2, RAD21, ITGB1, PPARG, NUSAP1, TNC, CCNB1, AXL, CDC2, BUB1, KITLG, IVNS1ABP, PEA15, NEUROG1, BTRC, IL11
Cell Death	2.36E-04	4.51E-13	ADM, HTR2B, BDNF, GNB1, HMOX1, GDF5, GMNN, UBE2V1, ANTXR2, AKT3, IFI6, CBFB, RHEB, MMP1, WEE1, RDX, CDK6, ITGA6, CIDEC, GREM1, ID3, TXNRD1, RASD1, ISG15, EMP1, IVNS1ABP, ADAM12, CXCL12, ZAK, CYR61, STEAP3, IL11	PLD2, CTGF, HTR2B, PLK3, HMMR, KRAS, TXNDC5, ODC1, CCNG1, GNB1, PKN2, MYLK, GDF5, EZR, VAMP3, CAV1, CBFB, ITGB5, SHISA5, RRM2, CIDEC, STAT3, ROR1, NDN, AREG, MAP3K12, CD9, BHLHB2, SNAI2, ZAK, THBS2, CYR61, TMEM158, STEAP3, ADM, BDNF, TPD52L1, DMD, INPP5A, EIF4E, VCAN, ID1, BBC3, ANTXR2, TOP2A, DAB2, IFI6, DUSP14, CTNNB1, FGF7, RAD21, CITED2, MMP1, ITGB1, PDZK1, CCNB1, TNC, GREM1, AXL, OAS3, F3, CYP1B1, CDC2, BUB1, KITLG, PLA2G4A, BMF, PEA15, PROCR, CD82, BTRC, ABCC3, PTGR1, ICMT, SMAD3, CSNK1A1, OLR1, CYFIP2, ITGAV, RHEB, DSP, TNFRSF11B, TUBB3, FST, THBS1, WEE1, RDX, CDK6, TXNRD1, RCAN1, RASD1, PDLIM4, NPC1, ADAM12, PTPN11, IGFBP3, LRDD, PMEPA1, PEBP1, SULF1, TPM1, ID2, RECK, HIF1A, NGF, TCF12, PDCD4, CIAPIN1, CCNA2, HMOX1, GMNN, GFRA1, UBE2V1, STAT1, MAGEH1, RAD23B, PPARG, TIMP3, MX1, FDXR, SERPINE2, EMP1, IVNS1ABP, PMP22, PRKCI, RRAS2, SATB1, DKK1, IL11
Cell Morphology	8.16E-04	9.90E-06	ADM, SLC14A1, BDNF, SPRY2, CXCL12, RDX, ITGA6, CDK6, ANXA2, CYR61, ID3	ICMT, RIT1, PLK3, SMAD3, KRAS, OLR1, ODC1, ROCK2, EZR, CAV1, ITGAV, CBFB, ITGB5, PODXL, FST, THBS1, FERMT2, CDK6, RDX, ANXA2, STAT3, ROR1, RCAN1, NDN, STC1, NPC1, MAP3K12, SDCBP, PTPN11, CD9, RND3, BHLHB2, SNAI2, THBS2, ZAK, IGFBP3, DHFR, FOXF1, CYR61, RAP2A, ADM, RAP1B, PEBP1, SULF1, TPM1, ID2, BDNF, RECK, NGF, EIF4E, VCAN, PDCD4, ID1, HMOX1, SH3D19, CCNA2, SIPA1, DAB2, NELF, ASPH, MARCKS, CTNNB1, FGF7, CITED2, RAD21, PPARG, ITGB1, TIMP3, TNC, AXL, CDC2, KITLG, PMP22, RRAS2, PRKCI, CAP2, CD82, DKK1, WNT5A
Cell Signaling	1.38E-03	ns	GNB1, ADM, TBCA, CXCL12, DIO2, RASD1, ABCA1, TXNRD1, OXTR	
Cell-To-Cell Signaling and Interaction	5.91E-06	2.38E-08	ADM, KCNN2, BDNF, RDX, ITGA6, GREM1, ANXA2, ID3, ABCA1, TXNRD1, STC1, HMOX1, MICB, ADAM12, CXCL12, SRXN1, TULP4, CYR61, MMP1, OXTR, IL11	C1ORF38, CTGF, KCNJ2, SMAD3, JAM2, OLR1, ABCA1, ROCK2, PKN2, CYFIP2, GDF5, CAV1, POSTN, ITGAV, ITGB5, TNFRSF11B, GNE, PODXL, THBS1, FERMT2, ANXA2, STAT3, TXNRD1, RCAN1, STC1, PTPN11, RND3, ADAM12, CD9, JAM3, NDUFA6, THBS2, IGFBP3, SRXN1, PDLIM1, CYR61, RAP2A, BDNF, ACTA2, PVRL3, HIF1A, NGF, VCAN, HMOX1, MICB, GFRA1, PDCL, SIPA1, DAB2, TULP4, CTNNB1, STAT1, MMP1, OXTR, ITGB1, PPARG, TIMP3, CCKAR, KCNN2, EPAS1, TNC, CDH6, GREM1, AXL, F3, NUDT15, KITLG, GCS1, CD82, ADAM9, IL11
Cellular Assembly and Organization	1.92E-03	6.68E-05	STC1, ADM, GNB1, BDNF, CXCL12, ITGA6, RDX, ANXA2, ABCA1, RASD1	KIF23, DLGAP5, CTGF, KCNJ2, SMAD3, NCAPG, CCNB2, KRAS, ROCK2, GNB1, EZR, CAV1, MYO10, DSP, THBS1, RDX, STAT3, EIF3E, ROR1, NDN, NPC1, PTPN11, CD9, RND3, CYR61, RAP1B, AKAP12, TPM1, BDNF, PICALM, RECK, PVRL3, NGF, DMD, INPP5A, VCAN, CCNA2, GFRA1, TOP2A, DAB2, NELF, MARCKS, FGF7, RAD21, PPARG, ITGB1, NUSAP1, CCKAR, CCNB1, TNC, AXL, CDC2, KITLG, PMP22, PRKCI, SERPINH1, CD82, KIF18A
Cellular Compromise	6.32E-03	7.66E-05	HMOX1, BDNF, CXCL12, RDX, ABCA1	ITGB1, CCNB1, THBS1, PDCL, ACTA2, ITGAV, CDC2
Cellular Development	1.39E-03	8.67E-09	AKAP12, YWHAG, BDNF, OSTM1, ITGA6, CDK6, GREM1, ANXA2, ID3, STC1, GDF5, SPRY2, CXCL12, NEUROG1, CBFB, CYR61, MMP1, IL11	CTGF, KCNJ2, RAB2A, KRAS, PPP1R14B, ODC1, PKN2, GDF5, EZR, IFITM2, CAV1, CBFB, HOOK1, ITGB5, BST1, YWHAG, ANXA2, STAT3, NDN, AREG, MAP3K12, CD9, SNAI2, BHLHB2, ZAK, THBS2, FOXF1, CYR61, AKAP12, BDNF, DMD, EIF4E, VCAN, ID1, TBX2, DAB2, UBE2D3, CTNNB1, FGF7, MMP1, ITGB1, TNC, SUZ12, GREM1, AXL, KITLG, CAP2, NEUROG1, CD82, ICMT, DLGAP5, AKR1C3, SMAD3, OLR1, ROCK2, VASH1, POSTN, ITGAV, DSP, TNFRSF11B, TUBB3, FST, THBS1, OSTM1, RDX, CDK6, RCAN1, STC1, IRF7, PTPN11, RND3, IGFBP3, RAP2A, PEBP1, TPM1, ID2, PICALM, KIAA0101, HIF1A, NGF, TCF12, IGLL1, CIAPIN1, HMOX1, GMNN, GFRA1, SIPA1, MARCKS, STAT1, MAGEH1, PPARG, EPAS1, IVNS1ABP, PMP22, PRKCI, RRAS2, SATB1, DKK1, IL11, WNT5A
Cellular Function and Maintenance	5.07E-03	4.82E-04	AKAP12, BICD2, BDNF, CXCL12, ACTR10	PICALM, ITGAV, CAV1, ITGB5
Cellular Growth and Proliferation	8.02E-04	1.17E-12	AKAP12, ADM, HTR2B, BTG3, BDNF, C9ORF78, GNB1, HMOX1, GDF5, AKT3, IFITM1, CBFB, OXTR, ADAMTS1, WEE1, ITGA6, CDK6, GREM1, ANXA2, ID3, RASD1, TXNRD1, ISG15, IVNS1ABP, STC1, EMP1, ADAM12, NT5E, CXCL12, SPRY2, NEUROG1, DHFR, RNF14, BAT2D1, CYR61, STEAP3, IL11	PLD2, CTGF, HTR2B, RAB2A, PLK3, HMMR, KRAS, ODC1, CCNG1, GNB1, GDF5, EZR, LEPRE1, CAV1, SERPINB7, CBFB, ITGB5, PDIA5, RRM2, ANLN, ANXA2, STAT3, NDN, AREG, CDC73, CD9, BHLHB2, SNAI2, THBS2, DHFR, FOXF1, MEST, CYR61, STEAP3, MAB21L1, ADM, RAP1B, AKAP12, BTG3, BDNF, DMD, ATP6AP1, EIF4E, VCAN, ID1, BBC3, DAB2, TBX2, IFITM1, UBE2D3, CTNNB1, FGF7, MMP1, CITED2, OXTR, ITGB1, CCKAR, PDZK1, CCNB1, TNC, SUZ12, GREM1, OAS3, AXL, CDC2, ISG15, BUB1, KITLG, PLA2G4A, SERPINH1, BMF, PEA15, NEUROG1, CD82, BTRC, RNF14, KIF23, WFDC1, DLGAP5, ICMT, AKR1C3, SMAD3, UBE2V2, ROCK2, POSTN, ITGAV, UBE2E3, DSP, SCN5A, TNFRSF11B, TUBB3, PAPPA, FST, ADAMTS1, THBS1, WEE1, CDK6, MYOF, TXNRD1, TPX2, RCAN1, RASD1, PDLIM4, SFRS3, STC1, ATP5B, ADAM12, PTPN11, IGFBP3, LRDD, HTRA1, FADS1, PMEPA1, DCBLD2, PEBP1, TPM1, DDX21, ID2, FRZB, EPB41L3, KIAA0101, RECK, C9ORF78, TAF7, HIF1A, NGF, TCF12, IGLL1, CCNA2, HMOX1, GFRA1, SIPA1, ENPP2, STAT1, PPARG, TIMP3, EPAS1, MX1, FDXR, SERPINE2, IVNS1ABP, EMP1, PMP22, PRKCI, NT5E, DKK1, WNT5A, IL11
Cellular Movement	4.08E-04	9.23E-11	ADM, BDNF, ITGA6, GREM1, ANXA2, ID3, GNB1, STC1, GDF5, ADAM12, CXCL12, SPRY2, SEMA6D, AKT3, NEUROG1, CBFB, CYR61, MMP1, IL11	KIF23, PLD2, CTGF, HMMR, SMAD3, CCNB2, OLR1, ODC1, GNB1, MYLK, ROCK2, VASH1, NARS, GDF5, EZR, ITGAV, POSTN, CAV1, CBFB, MYO10, ITGB5, TNFRSF11B, PODXL, FST, THBS1, FERMT2, ANLN, ANXA2, EIF3E, STAT3, NDN, AREG, STC1, NPC1, MAP3K12, SDCBP, RND3, PTPN11, ADAM12, CD9, JAM3, SNAI2, SEMA6D, THBS2, IGFBP3, CYR61, RAP2A, PEBP1, ADM, SULF1, TPM1, ID2, PICALM, BDNF, RECK, HIF1A, NGF, VCAN, PDCD4, ID1, GFRA1, TOP2A, ENPP2, MARCKS, STAT1, CTNNB1, FGF7, MMP1, ITGB1, PPARG, TIMP3, NUSAP1, CCKAR, CCNB1, TNC, GREM1, AXL, F3, CDC2, SERPINE2, IVNS1ABP, KITLG, PLA2G4A, PMP22, RRAS2, PRKCI, CCR4, ASAP2, PROCR, NEUROG1, CD82, SEMA3C, ADAM9, WNT5A, IL11
Cellular Response to Therapeutics	6.32E-03	ns	CYR61	
DNA Replication, Recombination, and Repair	6.32E-03	7.68E-04	NT5E, CXCL12	RAP1B, ADM, PEBP1, DLGAP5, NCAPG, CCNB2, NGF, ROCK2, CCNA2, ID1, TOP2A, CAV1, FGF7, RAD21, ITGB1, PPARG, NUSAP1, TNC, CCNB1, CDK6, GREM1, EIF3E, AREG, KITLG, RRAS2, PTPN11, THBS2, IGFBP3, EFEMP1, CYR61, KIF18A
Drug Metabolism	6.32E-03	ns	RDX, DHFR, ABCA1	
Energy Production	6.32E-03	ns	STC1	
Gene Expression	1.26E-02	2.91E-04	ITGA6, CBFB	HTR2B, SMAD3, KRAS, ATP8B1, PKN2, ITGAV, CAV1, CBFB, TEAD2, VPS4B, FST, YWHAZ, IRF9, STAT3, NDN, RCAN1, BACH1, IRF7, PTPN11, SNAI2, BHLHB2, PDLIM1, SMURF2, SNX6, FOXF1, RAP2A, AKAP12, PEBP1, DEK, ID2, BDNF, TAF7, HIF1A, NGF, ZBTB38, PDCD4, TCF12, CCNA2, ID1, UBE2V1, TOP2A, DAB2, TBX2, STAT1, CTNNB1, FGF7, CITED2, PPARG, ITGB1, TRIP13, JAZF1, CCNB1, EPAS1, SUZ12, CDC2, SERPINE2, IVNS1ABP, HTATSF1, PRKCI, SATB1, PEA15, IGF2BP2, NEUROG1, LBH, DKK1, RNF14, TFB1M, HLTF, IL11
Lipid Metabolism	1.73E-03	1.53E-03	ADM, RDH11, CXCL12, ABCA1	KITLG, ROCK2, PLA2G4A, NPC1, CD9, CAV1, ENPP2, CD82, HIF1A, STAT3, NGF, ABCA1
Molecular Transport	1.73E-03	3.35E-06	ADM, RDH11, BDNF, RDX, SLC39A14, DIO2, ABCA1, TXNRD1, STC1, NT5E, SLC14A1, CXCL12, NEDD4L, STEAP3	ITGB1, STAT3, HIF1A, NGF, ABCA1, ROCK2, KITLG, NPC1, PLA2G4A, SATB1, IGFBP3, CAV1, ITGAV, ENPP2
Nucleic Acid Metabolism	1.38E-03	ns	GNB1, ADM, HMOX1, NT5E, CXCL12, UAP1, RASD1, ABCA1, OXTR	
Post-Translational Modification	1.26E-02	6.87E-05	ADM, CXCL12, GREM1, ANXA2	ADM, ICMT, ZDHHC2, PLD2, EPB41L3, PTPLA, UBE2V2, NGF, ODC1, ABCA1, GALNAC4S-6ST, MYLK, PDCL, UBE2V1, ITGAV, FBXO11, UBE2D3, RNF144B, STAT1, FGF7, MMP1, ITGB1, PDIA5, MOBKL1A, TBCA, SUZ12, THBS1, ATG3, CDK6, GREM1, SEP15, CDC2, ISG15, KITLG, PPP2CB, MAP3K12, WHSC1L1, PTPN11, CD9, SATB1, GCS1, IGFBP3, ATXN10
Protein Folding	1.26E-02	ns	TBCA	
Protein Trafficking	ns	3.35E-06		ITGB1, SATB1, IGFBP3, ITGAV

Small Molecule Biochemistry	1.38E-03	4.82E-04	ADM, RDH11, RDX, ANXA2, DIO2, ABCA1, RASD1, GFPT2, GNB1, HMOX1, NT5E, CXCL12, UAP1, DHFR, OXTR	GLS, STAT3, HIF1A, NGF, ABCA1, GFPT2, ROCK2, KITLG, NPC1, PLA2G4A, CD9, CAV1, CD82, ENPP2
Vitamin and Mineral Metabolism	6.32E-03	ns	RDH11, DHFR, DIO2, ABCA1	
