# Supplementary material for: Identification of Keratinocyte Growth Factor as a Target of microRNA-155 in Lung Fibroblasts: Implication in Epithelial-Mesenchymal Interactions
Source: PLoS One. 2009 Aug 24;4(8):e6718. doi: 10.1371/journal.pone.0006718 (PMC2726943; doi:10.1371/journal.pone.0006718)
Supplement: Table S3 — Full list of the miR-155 predicted targets down-regulated following miR-155 overexpression in HFL1. The 260 transcripts predicted to be miR-155 targets by at least one of the following algorithm: TargetScan, Pictar and MicroCible, are listed. Logarithm (base 2) of the ratio of miR-155/miR-Neg and false discovery rate p-values using the Benjamini-Hochberg correction are represented. ID: correspond to RNG oligo IDs that give access to transcripts and probes annotations through our system of information Mediante (http://www.microarray.fr:8080/merge/index). (0.10 MB PDF) [file pone.0006718.s005.pdf]

N°	Name	ID	UNIGENE_ID	ACCESSION_NUMBER	log2 Ratio		adj.P.Val	
					24h	48h	24h	48h
1	ACTR10	34236	Hs.509451	NM_018477	-1.41	-2.01	3.66E-06	3.89E-07
2	ACVR1	90666	Hs.470316	NM_001105	-0.04	-0.81	8.31E-01	1.73E-04
3	ADAMTS1	16014	Hs.534115	NM_006988	-1.15	-2.26	1.01E-05	5.42E-07
4	ADRB2	83352	Hs.591251	NM_000024	-0.83	-0.61	6.86E-05	1.33E-04
5	ANKMY2	103133	Hs.157378	NM_020319	-0.86	-1.15	3.77E-04	5.00E-05
6	ANTXR2	49597	Hs.162963	NM_058172	-1.30	-1.54	1.49E-05	3.82E-06
7	ANXA2	7407	Hs.511605	NM_001002857;NM_004039;NM_001002858;NR_001446	-0.58	-1.24	1.97E-01	7.41E-03
	ANXA2	132294	Hs.511605	NM_001002857;NM_004039;NM_001002858;NR_001446	-1.18	-2.67	2.74E-03	1.53E-05
8	ANXA3	8955	Hs.480042	NM_005139	-1.18	-2.94	6.54E-03	2.48E-05
9	AP1B1	104373	Hs.368794	NM_001127;NM_145730	-0.69	-0.43	3.38E-05	1.46E-04
10	ARL2BP	21287	Hs.513631	NM_012106	-1.94	-2.61	1.36E-04	9.74E-06
11	ARL6IP5	131104	Hs.518060	NM_006407	-0.01	-0.81	9.39E-01	3.98E-05
12	ARPC3	9688	Hs.524741	NM_005719	-0.65	-0.99	3.58E-05	1.52E-06
13	ASPH	105511	Hs.332422	NM_032468;NM_032466	-0.88	-1.73	7.12E-05	1.52E-06
	ASPH	105515	Hs.332422	NM_032468;NM_032466	-0.55	-1.66	6.56E-03	9.31E-06
14	ATG3	39274	Hs.477126	NM_022488	-0.80	-1.19	1.97E-04	2.38E-05
15	ATP10D	36649	Hs.437241	NM_020453	-1.44	-1.54	1.13E-04	4.53E-05
16	ATP6V1D	25500	Hs.272630	NM_015994	-0.83	-0.67	7.44E-05	1.93E-04
17	ATXN10	21696	Hs.475125	NM_013236	-1.11	-1.08	3.07E-04	1.80E-04
18	BACH1	90782	Hs.154276	NM_001186;NM_206866	-0.85	-1.12	2.78E-04	4.75E-05
19	BDKRB2	90051	Hs.525572	NM_000623	-0.96	-0.55	1.01E-03	1.30E-02
20	BDNF	91429	Hs.502182	NM_170734;NM_001709;NM_170731;NM_170733;NM_170735;NM_170732	-1.15	-1.72	8.59E-03	4.55E-04
21	BIRC3	87599	Hs.127799	NM_182962;NM_001165	-0.56	-0.35	5.82E-04	4.31E-03
22	BTBD1	45748	Hs.459149	NM_025238;NM_001011885	0.13	-0.59	1.49E-01	7.26E-05
23	BTBD3	26343	Hs.244590	NM_181443;NM_014962	-0.91	-1.22	3.47E-05	4.35E-06
24	BTBD7	30580	Hs.525549	NM_018167	-0.33	-0.52	1.71E-03	9.53E-05
25	CALU	89284	Hs.643549	NM_001219	-0.95	-1.21	3.66E-04	3.50E-05
26	CAMTA1	77481	Hs.397705	AY037153	-0.55	-1.17	3.87E-03	5.35E-05
27	CARHSP1	22307	Hs.632184	NM_014316;NM_001042476	-0.22	-0.65	1.43E-02	2.94E-05
28	CASP6	168755	Hs.389452	NM_032992;NM_001226	-0.25	-0.79	2.15E-02	6.75E-05
29	CASP9	87679	Hs.329502	NM_032996;NM_001229	-0.55	-0.51	1.16E-04	1.91E-04
30	CBFB	94541	Hs.460988	NM_022845;NM_001755	-1.57	-1.74	1.03E-03	3.46E-04
31	CCDC41	25679	Hs.279209	NM_001042399;NM_016122	-0.48	-0.51	3.42E-04	9.70E-05
32	CCT2	111218	Hs.189772	NM_006431	-0.07	-0.87	7.11E-01	2.14E-04
33	CDC73	38344	Hs.576497	NM_024529	-0.66	-1.03	5.01E-03	2.50E-04
34	CDCA7	46388	Hs.470654	NM_031942;NM_145810	-0.92	-0.54	2.16E-05	1.33E-04
35	CDV3	29683	Hs.642726	NM_017548	-1.24	-2.00	5.40E-03	2.37E-04
36	CEP63	42494	Hs.443301	NM_025180;NM_001042384;NM_001042383;NM_001042400	-0.22	-0.61	2.94E-02	1.45E-04

37	CEP70	39884	Hs.531962	NM_024491	-0.51	-0.62	3.30E-03	5.88E-04
38	CFL2	139860	Hs.180141	NM_138638;NM_021914	-0.79	-1.83	1.20E-02	9.10E-05
39	CHCHD7	39808	Hs.436913	NM_001011671;NM_001011669;NM_001011670;NM_001011668;NM_024300;NM_0010 11667	-0.28	-0.74	5.12E-02	2.58E-04
40	CHSY1	23060	Hs.110488	NM_014918	-1.21	-1.40	8.04E-06	2.68E-06
41	CIAPIN1	163555	Hs.4900	NM_020313	-0.64	-1.08	3.28E-05	2.74E-06
42	CLCN3	93105	Hs.481186	NM_173872;NM_001829	-0.70	-0.96	2.74E-03	1.98E-04
43	CNIH	12884	Hs.294603	NM_005776;NM_001009551	-0.39	-1.77	6.72E-02	4.74E-05
44	COPS3	97088	Hs.6076	NM_003653	-0.53	-0.84	2.12E-03	6.91E-05
45	CPD	92443	Hs.446079	NM_001304	-1.06	-2.05	1.08E-05	5.42E-07
46	CREG1	7220	Hs.5710	NM_003851	-0.15	-0.98	1.16E-01	1.23E-05
47	CSNK1A1	91649	Hs.529862	NM_001025105;NM_001892	-0.98	-1.29	1.53E-05	1.52E-06
48	CUGBP2	17048	Hs.309288	NM_001025077;NM_001083591;NM_001025076;NM_006561	-0.60	-1.17	1.33E-02	2.35E-04
49	CYFIP1	22658	Hs.26704	NM_014608;NM_001033028	-0.72	-1.00	1.85E-03	2.13E-04
50	CYR61	94295	Hs.8867	NM_001554	-1.38	-2.81	9.13E-03	9.18E-05
51	DCUN1D3	155818	Hs.101007	NM_173475	-0.69	-0.49	3.12E-04	4.96E-04
52	DCUN1D4	84291	Hs.221407	NM_001040402;NM_015115	-1.05	-1.23	7.66E-06	2.24E-06
53	DDEF2	5660	Hs.555902	NM_003887	-0.95	-1.30	3.31E-04	3.03E-05
54	DEK	99927	Hs.484813	NM_003472	-1.11	-1.57	3.95E-04	5.39E-05
55	DNAJC15	21703	Hs.438830	NM_013238	-0.30	-0.79	5.95E-03	1.45E-05
56	DNAJC19	58938	Hs.230601	NM_145261;NM_201259;NM_201261;NM_201260	-1.03	-1.43	3.69E-04	3.61E-05
	DNAJC19	58939	Hs.230601	NM_145261;NM_201259	-0.96	-1.39	1.27E-04	7.05E-06
57	DNTTIP1	50999	Hs.472852	NM_052951	-0.34	-0.95	1.54E-03	9.42E-06
58	DPY19L1	211	Hs.408623	AB020684;NM_015283	-2.55	-2.66	3.46E-05	1.55E-05
59	DUSP14	16060	Hs.91448	NM_007026	-0.61	-1.31	1.46E-03	2.06E-05
60	DYRK3	100025	Hs.164267	NM_001004023;NM_003582	-0.75	-0.54	8.21E-04	3.33E-03
61	ECHDC1	34245	Hs.486410	NM_001105545;NM_001105544;NM_001002030;NM_018479	0.00	-0.57	9.96E-01	4.05E-04
62	ELF4	94119	Hs.271940	NM_001421	-0.55	-0.35	7.07E-04	6.58E-03
63	ELL2	21259	Hs.592742	NM_012081	-1.38	-2.83	7.07E-04	9.31E-06
64	EPAS1	100922	Hs.468410	NM_001430;AA680300	-0.66	-1.27	2.63E-02	6.28E-04
65	FAM104A	50303	Hs.103555	NM_001098832;NM_032837	-0.24	-0.55	9.92E-03	1.62E-04
66	FAM18B	116411	Hs.87295	NM_016078	-0.08	-0.62	5.48E-01	3.13E-04
67	FAM29A	28153	Hs.533468	NM_017645	-0.69	-0.46	3.49E-04	1.46E-03
68	FAM3C	116003	Hs.434053	NM_001040020;NM_014888	-0.45	-1.23	5.04E-02	1.76E-04
69	FBXO11	169922	Hs.352677	NM_025133	-0.93	-1.05	2.70E-04	6.55E-05
70	FBXO30	44964	Hs.421095	NM_032145	-0.17	-0.57	9.72E-02	2.05E-04
71	FEM1C	33240	Hs.47367	NM_020177	-0.36	-0.52	7.08E-03	7.09E-04
72	FEZ2	8900	Hs.258563	NM_005102;NM_001042548	-1.60	-2.09	4.70E-06	6.97E-07
73	FGF7	129551	Hs.567268	NM_002009	-0.78	-1.33	2.71E-03	9.21E-05
	FGF7	172697	Hs.567268	NM_002009;AK054997	-1.12	-1.55	6.86E-04	3.12E-05

74	FLJ13611	45386	Hs.591760	NM_024941;NM_001093756;NM_001093755	-0.69	-0.65	3.05E-04	1.41E-04
75	FLJ20147	29852		NM_017687	-1.19	-0.67	5.23E-06	1.83E-05
76	FNDC3B	37840	Hs.159430	NM_022763	-0.35	-0.87	6.68E-03	4.69E-05
77	FRYL	74114		NM_015030	-0.45	-1.08	3.54E-03	3.30E-05
78	FYTTD1	48209	Hs.277533	NM_001011537;NM_032288	-1.15	-1.37	2.16E-05	5.54E-06
	FYTTD1	48212	Hs.277533	NM_001011537;NM_032288	-0.66	-1.29	3.54E-02	6.43E-04
79	GALT	107581	Hs.522090	NM_147131;NM_000155;NM_147132	-0.77	-0.98	2.65E-05	8.13E-06
80	GBA2	36858	Hs.443134	NM_020944	-0.61	-0.45	1.86E-03	5.59E-03
81	GFRA1	10643	Hs.591913	NM_005264;NM_145793	-0.70	-1.60	8.19E-03	8.72E-05
82	GHITM	140070	Hs.352656	NM_014394	0.01	-0.86	9.60E-01	2.59E-04
83	GLT25D1	41747	Hs.418795	NM_024656	-0.22	-0.91	4.97E-02	3.62E-05
84	GMNN	26968	Hs.234896	NM_015895	-1.80	-1.84	3.27E-05	1.82E-05
85	GOLT1B	25610	Hs.62275	NM_016072	-0.84	-0.87	8.76E-05	5.21E-05
86	GOT1	93452	Hs.500756	NM_002079	-0.70	-1.19	2.98E-05	1.06E-06
87	GPR126	72392	Hs.318894	NM_001032394;NM_198569;NM_001032395;NM_020455	-1.43	-2.02	3.66E-04	2.65E-05
88	GPSM2	21768	Hs.584901	NM_013296	-0.88	-0.60	5.38E-04	3.22E-03
89	GRPEL1	42507	Hs.443723	NM_025196	-0.51	-0.84	1.37E-03	3.50E-05
90	GYG1	4403	Hs.477892	NM_004130	-1.09	-1.32	2.76E-05	3.77E-06
91	H3F3A	91945	Hs.546259	NM_002107	-1.73	-2.31	3.64E-04	2.61E-05
92	HADHB	89454	Hs.534639	NM_000183	-0.30	-0.63	8.63E-03	8.79E-05
93	HBP1	19881	Hs.162032	NM_012257	-0.64	-0.26	1.71E-03	8.34E-02
94	HDHD2	44923	Hs.465041	NM_032124	-1.50	-2.01	3.66E-06	2.72E-07
95	HELZ	26237	Hs.631739	NM_014877	-0.21	-0.62	5.55E-02	1.98E-04
96	HIF1A	91228	Hs.509554	NM_001530	-0.05	-1.02	8.81E-01	6.88E-04
	HIF1A	174919	Hs.509554	NM_001530;NM_181054	0.07	-0.74	8.54E-01	1.21E-02
97	HMGB3	12326	Hs.19114	NM_005342	-0.69	-0.64	1.56E-03	1.14E-03
98	HNRPA3	164571	Hs.516539	NM_005758;NM_194247	-1.81	-2.21	7.61E-05	1.09E-05
99	HSD17B12	24071	Hs.132513	NM_016142	-1.29	-2.06	7.60E-04	4.61E-05
100	HTATSF1	115350	Hs.204475	NM_014500	0.14	-1.05	4.60E-01	2.09E-04
101	HTR2B	88845	Hs.421649	NM_000867	-1.57	-3.45	4.83E-04	3.96E-06
102	IER5	137631	Hs.15725	NM_016545	-0.73	-0.50	1.58E-05	9.64E-05
103	IFNAR1	130256	Hs.529400	NM_000629	0.03	-0.53	8.72E-01	6.46E-04
104	IGFBP5	81532	Hs.369982	BC018427;NM_000599	-0.13	-0.89	5.35E-01	5.18E-04
105	INPP5A	12584	Hs.523360	NM_005539	-1.19	-1.30	1.07E-05	1.93E-06
106	KCNN2	173651	Hs.98280	NM_021614;NM_170775	-1.40	-2.51	9.41E-06	5.42E-07
107	KCTD3	24048	Hs.335139	NM_016121	-0.93	-1.32	8.04E-06	9.96E-07
108	KCTD9	31421	Hs.72071	NM_017634	-0.52	-1.18	4.99E-02	5.01E-04
109	KITLG	162205	Hs.1048	AK055903;NM_003994;NM_000899	-0.56	-1.61	1.91E-02	6.39E-05
110	KRCC1	29475	Hs.469254	NM_016618	-0.43	-0.70	1.68E-03	3.61E-05
111	LDLRAP1	23624	Hs.590911	NM_015627	-0.41	-0.59	7.44E-04	5.96E-05

112	LHFPL2	82990	Hs.79299	NM_005779	-0.54	-0.67	1.21E-03	2.16E-04
113	LKAP	36188	Hs.173524	NM_019081;NM_014647	-0.69	-0.47	2.27E-04	1.12E-03
114	LOC400657	160843	Hs.61508	NM_001008234	-0.43	-0.57	1.38E-03	1.87E-04
115	LOC644246	81628	Hs.463231	BC020847	-0.77	-0.84	3.21E-05	1.86E-05
116	LPGAT1	26227	Hs.591421	NM_014873	-0.91	-1.33	3.27E-05	2.41E-06
117	LRCH2	35301	Hs.65366	NM_020871	-0.57	-0.60	1.91E-03	7.62E-04
118	LRRC17	119965	Hs.567412	NM_005824;NM_001031692	0.14	-0.60	5.06E-02	1.30E-05
119	LRRC42	47910	Hs.40094	NM_052940	-0.66	-0.94	1.23E-03	6.92E-05
	LRRC42	47914	Hs.40094	NM_052940	-0.29	-0.88	2.14E-01	1.76E-03
120	LRRC59	32621	Hs.370927	NM_018509	-0.96	-0.98	6.91E-04	2.12E-04
121	LRRTM4	137992	Hs.285782	NM_024993	-0.37	-0.69	8.97E-03	2.77E-04
122	LY6K	28041	Hs.69517	NM_017527	-1.43	-1.56	9.63E-06	2.74E-06
123	LYCAT	162784	Hs.468048	NM_001002257;NM_182551	-0.65	-0.80	1.52E-04	3.50E-05
124	MAP3K7IP2	163253	Hs.269775	NM_145342;NM_015093	-1.26	-1.34	1.52E-04	1.81E-04
125	MARCH6	131379	Hs.432862	NM_005885	-0.18	-0.51	6.60E-02	5.69E-04
126	MARVELD2	55574	Hs.444195	NM_144724	-0.48	-0.82	7.43E-05	8.39E-06
127	MCPH1	38457	Hs.490892	NM_024596	-0.39	-0.68	2.63E-03	1.08E-04
128	MEGF6	149280	Hs.593645	AF086414;NM_001409	-0.87	-0.73	6.14E-04	5.71E-04
129	MEIS1	95402	Hs.56186<br>Hs.526754	NM_002398	-0.99	-0.76	3.64E-04	1.02E-03
130	MEST	93841	Hs.270978	NM_177524;NM_177525;NM_002402	-0.57	-2.23	6.97E-05	1.48E-07
131	MGC16169	47509	Hs.292986	NM_033115	-1.10	-0.82	4.09E-05	3.50E-05
132	MGST1	178418	Hs.389700	NM_145764;NM_145791;NM_020300;NM_145792	-0.04	-0.73	8.66E-01	3.36E-04
133	MMD	19979	Hs.463483	NM_012329	-1.26	-1.82	6.20E-03	3.84E-04
134	MOCS2	6487	Hs.163645	NM_176806;NM_183418;NM_004531	-0.75	-1.61	1.53E-04	1.52E-06
135	MORC3	105283	Hs.421150	NM_015358	-0.41	-0.81	1.77E-03	1.61E-05
136	MPI	92346	Hs.75694	NM_002435	-0.90	-1.21	3.90E-04	5.41E-05
137	MRPL18	19031	Hs.416998	NM_014161	-0.62	-1.02	3.76E-03	1.65E-04
138	MRPS27	26419	Hs.482491	NM_015084	-0.67	-0.95	3.12E-04	2.25E-05
139	MTMR6	6713	Hs.507536	NM_004685	-0.33	-0.75	1.97E-02	2.17E-04
140	MYO10	18351	Hs.481720	NM_012334	-0.89	-1.95	1.06E-02	1.20E-04
141	NARS	8092	Hs.465224	NM_004539	-0.96	-1.25	2.41E-05	6.42E-06
142	NAT13	44026	Hs.269528	NM_025146	-1.06	-1.69	1.05E-04	3.52E-06
143	NAV3	116703	Hs.306322	NM_014903	-0.04	-0.58	6.21E-01	3.50E-05
144	NECAP1	26725	Hs.555927	NM_015509	-0.68	-1.06	5.64E-04	3.50E-05
145	NOLA1	144781	Hs.69851	NM_018983;NM_032993	-0.66	-1.05	1.10E-03	6.61E-05
146	NR2F2	35399	Hs.347991	NM_021005	-0.15	-0.62	1.55E-01	1.81E-04
147	NT5E	98661	Hs.153952	NM_002526	-1.03	-2.84	1.08E-04	7.83E-07
148	NTF3	95566	Hs.99171	NM_001102654;NM_002527	-0.41	-0.83	1.61E-02	2.05E-04
149	OBFC2A	39466	Hs.591610	NM_022837;NM_001031716	-1.20	-2.67	2.93E-04	2.35E-06

150	OSBPL11	41115	Hs.477440	NM_022776	-0.44	-0.71	3.17E-03	1.38E-04
151	OSBPL8	35282	Hs.430849	NM_001003712;NM_020841	-0.24	-0.78	1.89E-02	2.75E-05
152	OSTM1	18875	Hs.226780	NM_014028	-1.51	-2.02	1.78E-03	1.34E-04
153	OXCT1	88233	Hs.278277	NM_000436	-0.69	-0.76	2.98E-05	4.72E-06
154	PALLD	25632	Hs.151220	NM_016081	-0.48	-0.58	9.27E-04	2.17E-04
155	PAPOLA	48544	Hs.253726	NM_032632	-0.63	-1.01	3.32E-05	4.01E-06
156	PAWR	95633	Hs.406074	NM_002583	-0.53	-0.84	4.02E-04	2.16E-05
157	PCCA	89612	Hs.80741	NM_000282	-0.60	-0.59	1.73E-04	5.86E-05
158	PCDH18	36138	Hs.591691	NM_019035	-1.27	-0.88	9.97E-05	2.00E-04
159	PDHA1	89618	Hs.530331	NM_000284	-0.35	-0.55	2.66E-03	1.86E-04
160	PDS5A	24881	Hs.331431	NM_001100399;NM_015200	-0.14	-0.52	1.84E-01	6.34E-04
161	PEA15	5529	Hs.517216	NM_003768	-0.73	-1.54	2.03E-02	2.34E-04
162	PEBP1	97200	Hs.433863	NM_002567	-0.76	-1.39	3.24E-03	4.40E-05
163	PERP	40750	Hs.520421	NM_022121	-0.03	-0.58	8.74E-01	5.99E-04
164	PHACTR2	26018	Hs.102471	NM_001100164;NM_001100166;NM_001100165;NM_014721	-1.16	-2.03	1.14E-02	2.74E-04
165	PMP22	107586	Hs.372031	NM_153322;NM_153321;NM_000304	-0.90	-1.92	2.59E-03	2.41E-05
166	PODXL	12409	Hs.16426	NM_005397;NM_001018111	-0.58	-1.44	3.78E-03	1.88E-05
167	POLE3	29616	Hs.108112	NM_017443	-1.61	-1.71	3.66E-06	6.18E-07
168	PPP2CB	5983	Hs.491440	NM_001009552;NM_004156	-0.95	-2.62	3.47E-05	5.66E-07
169	PRDM5	127629	Hs.132593	NM_018699	-0.43	-0.79	8.08E-03	2.52E-04
170	PRKCI	97410	Hs.478199	NM_002740	-0.74	-1.10	9.05E-03	6.92E-04
171	PRNP	88018	Hs.472010	NM_000311;NM_183079;NM_001080121;NM_001080123;NM_001080122	-0.27	-0.93	5.24E-02	5.76E-05
172	PRPF38A	163434	Hs.5301	NM_032864	-0.11	-0.68	4.10E-01	2.13E-04
173	PRR16	31194	Hs.157461	NM_016644	-0.43	-0.84	2.01E-02	6.18E-04
174	PRRG1	87342	Hs.190341	NM_000950	-0.17	-0.60	1.21E-01	8.99E-05
175	PRSS35	62024	Hs.98381	NM_153362	-1.13	-1.82	2.24E-03	2.13E-04
176	PSKH1	109612	Hs.513683	NM_006742	-1.09	-0.59	1.19E-03	1.96E-02
177	PTPN11	50403	Hs.506852	NM_002834	-0.36	-1.32	3.35E-02	2.08E-05
178	PUS7L	44546	Hs.445814	NM_031292;NM_001098614;NM_001098615	-0.56	-0.62	1.76E-03	4.15E-04
179	RAB11FIP2	24644	Hs.173656	NM_014904	-1.37	-1.50	2.92E-04	8.41E-05
180	RAB34	46380	Hs.301853	NM_031934	-0.76	-0.88	1.29E-03	5.04E-04
181	RAB6A	46558	Hs.503222<br>Hs.591552	NM_002869;NM_198896;NM_001077637;NM_032144	-0.64	-0.79	1.04E-03	1.02E-04
	RAB6A	96036	Hs.503222<br>Hs.591552	NM_198896;NM_002869;NM_001077637;NM_032144	-0.73	-0.90	2.22E-04	4.55E-05
182	RAC1	108740	Hs.413812	NM_006908;NM_018890;NM_198829	-0.17	-0.92	4.35E-01	6.46E-04
183	RAD21	15081	Hs.81848	NM_006265	-0.39	-1.22	7.47E-03	1.44E-05
184	RAI14	26785	Hs.431400	NM_015577	-0.77	-1.67	2.16E-05	6.68E-07
185	RAP1B	26844	Hs.369920	NM_001089704;NM_015646;NM_001010942	-0.14	-1.15	1.68E-01	1.36E-05
186	RAP2A	33842	Hs.508480	NM_021033	-0.68	-1.62	1.35E-02	6.24E-05
187	RCN2	96066	Hs.79088	NM_002902	-1.49	-2.48	2.88E-03	8.04E-05

188	RECK	118772	Hs.388918	NM_021111	-0.68	-1.72	9.48E-03	3.99E-05
189	RHEB	9548	Hs.438823	NM_005614	-1.57	-2.00	7.39E-06	9.94E-07
190	RND3	126716	Hs.6838	NM_005168	0.50	-1.40	1.22E-02	3.21E-05
191	RNF135	46770	Hs.29874	NM_032322;NM_197939	-0.58	-0.64	1.19E-03	4.96E-04
192	RNF26	43267	Hs.524084	NM_032015	-0.63	-0.38	8.89E-04	9.46E-03
193	RPL17	130114	Hs.374588	NM_000985	-0.54	-1.55	1.52E-02	5.00E-05
194	S100PBP	41076	Hs.440880	NM_022753	-0.65	-0.73	7.73E-04	1.47E-04
195	SACM1L	18867	Hs.156509	NM_014016	-0.78	-0.83	7.43E-05	2.80E-05
196	SATB1	97734	Hs.517717	NM_002971	-0.59	-1.06	6.23E-03	1.85E-04
197	SCN9A	96164	Hs.2319	NM_002977	-0.57	-0.87	2.44E-04	1.74E-05
	SCN9A	170806	Hs.2319	NM_002977;AJ310897	0.11	-0.45	5.89E-01	1.41E-02
198	SDCBP	11180	Hs.200804	NM_001007068;NM_001007069;NM_001007070;NM_005625;NM_001007067	-0.99	-2.66	1.42E-03	3.26E-06
199	SELT	27460	Hs.369052	NM_016275	0.05	-0.84	7.67E-01	1.26E-04
200	SERTAD2	24448	Hs.591569	NM_014755	-0.69	-0.55	3.81E-04	1.24E-03
201	SESTD1	80632	Hs.591613	NM_178123	-0.92	-0.59	8.04E-06	3.80E-05
202	SGK3	20197	Hs.545401	NM_001033578;NM_170709;NM_013257	-0.63	-0.59	1.20E-03	7.22E-04
203	SH3BP4	22586	Hs.516777	NM_014521	-0.75	-1.73	8.17E-03	4.45E-05
204	SKIV2L2	24972	Hs.274531	NM_015360	-0.66	-1.22	5.67E-05	3.71E-06
205	SLC20A2	14142	Hs.491611	NM_006749	-0.61	-1.16	6.56E-03	8.99E-05
206	SLC35F5	45676	Hs.632527	NM_025181	-0.70	-1.90	2.89E-02	8.41E-05
207	SLC38A1	42656	Hs.533770	NM_001077484;NM_030674	-0.14	-0.82	3.28E-01	5.16E-04
208	SLFN11	62475	Hs.462829	NM_001104589;NM_152270;NM_001104588;NM_001104590;NM_001104587	-0.89	-0.37	5.89E-04	3.58E-02
209	SMAD3	9939	Hs.36915	NM_005902	-0.13	-0.51	1.98E-01	2.21E-04
210	SMURF2	37800	Hs.515011	NM_022739	-0.32	-1.39	2.77E-02	1.26E-05
211	SMYD2	33263	Hs.66170	NM_020197	-0.31	-1.24	3.56E-02	2.37E-05
212	SOCS6	111250	Hs.591068	NM_004232;AF161545	-0.65	-0.94	7.43E-05	7.34E-06
213	SPIRE1	66320	Hs.515283	NM_020148	-0.84	-0.97	8.43E-05	2.03E-05
214	SRXN1	56000	Hs.516830	NM_080725	-1.04	-1.80	6.06E-03	1.42E-04
215	SSX2IP	20466	Hs.22587	NM_014021	-0.90	-0.57	1.04E-03	4.63E-03
216	STRN3	22620	Hs.401843	NM_001083893;NM_014574	-0.48	-0.78	1.59E-02	6.55E-04
217	SUCLG2	73984	Hs.186512	NM_003848	-0.18	-0.98	3.12E-02	4.19E-06
218	SUZ12	129955	Hs.462732	NM_015355	-0.08	-1.50	7.08E-01	6.24E-05
219	TAF7	11204	Hs.438838	NM_005642	-0.86	-1.58	1.71E-04	4.28E-06
	TAF7	11208	Hs.438838	NM_005642	-0.18	-0.92	6.49E-01	7.15E-03
220	TBCA	8185	Hs.291212	NM_004607	-1.53	-1.78	7.81E-05	1.40E-05
221	TCF12	96485	Hs.511504	NM_207036;NM_207037;NM_003205;NM_207038;NM_207040	-0.43	-1.02	1.75E-03	1.47E-05
222	TERF1	178729	Hs.442707	NM_003218;NM_017489	-0.78	-0.65	1.91E-04	3.93E-04
223	TMED5	27172	Hs.482873	NM_016040	-0.23	-0.98	1.61E-01	2.22E-04
224	TMEM33	127309	Hs.31082	NM_018126	-1.28	-1.71	1.87E-04	2.03E-05
225	TNS3	39358	Hs.520814	NM_022748	-0.69	-0.91	5.33E-04	5.76E-05

226	TOMM20	143603	Hs.533192	NM_014765	-0.67	-0.86	3.50E-04	3.26E-05
227	TPRKB	27191	Hs.157401	NM_016058	-1.29	-1.28	3.90E-04	2.15E-04
228	TRAM1	19201	Hs.491988	NM_014294	-2.89	-4.20	1.46E-03	8.41E-05
229	TRIP13	6094	Hs.436187	NM_004237	-1.49	-1.58	7.66E-06	1.63E-06
230	TSGA14	34406	Hs.368315	NM_018718	-1.03	-1.29	2.08E-05	3.96E-06
231	TSHZ3	36819	Hs.278436	NM_020856	-0.84	-0.92	1.62E-03	4.30E-04
232	TSPAN14	42822	Hs.310453	NM_030927	-1.04	-0.76	1.08E-05	6.68E-06
233	TULP4	34950	Hs.486993	NM_001007466;NM_020245	-1.44	-1.25	7.66E-06	8.66E-06
234	TXNDC12	25401	Hs.476033	NM_015913	-1.55	-1.42	2.06E-05	9.31E-06
235	UBE2D3	99746	Hs.518773	NM_181891;NM_181887;NM_181888;NM_181892;NM_181886;NM_181893;NM_181890;NM_003340;NM_181889	-0.53	-1.73	5.64E-04	2.17E-06
	UBE2D3	142694	Hs.518773	NM_181888;NM_181891;NM_181887;NM_181892;HSM804727;NM_181886;NM_181893;NM_181890;NM_003340;NM_181889	-0.42	-1.96	1.90E-03	1.06E-06
236	UBL3	16141	Hs.145575	NM_007106	-0.27	-0.59	6.69E-03	1.93E-04
237	USP33	24777	Hs.480597	NM_015017;NM_201624	-0.54	-0.80	3.62E-04	1.99E-05
238	USP48	46651	Hs.467524	NM_032236	-0.53	-0.33	1.54E-03	1.67E-02
239	USP8	8980	Hs.569446	NM_005154	-0.55	-0.79	2.02E-03	1.03E-04
240	USP9X	107904	Hs.77578	NM_001039591;NM_001039590	-0.28	-0.57	9.71E-03	2.31E-04
241	VAMP3	5265	Hs.66708	NM_004781	-0.72	-1.34	2.69E-02	6.09E-04
242	VEZF1	148281	Hs.463569	AK058024;NM_007146	-0.75	-0.60	6.06E-05	1.13E-04
243	VGLL3	24138	Hs.435013	NM_016206	-0.83	-1.30	1.74E-03	8.29E-05
244	VPS4B	5406	Hs.126550	NM_004869	-0.85	-1.17	1.12E-03	9.54E-05
245	WDFY1	33696	Hs.368359	NM_020830	-0.62	-1.38	9.59E-03	9.92E-05
246	WDHD1	17707	Hs.385998	NM_001008396;NM_007086	-0.39	-0.58	7.07E-04	4.33E-05
247	WEE1	98284	Hs.249441	NM_003390	-1.09	-1.25	2.64E-03	6.63E-04
248	WHSC1L1	109350	Hs.608111	NM_017778	-1.00	-1.17	4.75E-04	1.31E-04
249	WTAP	169662	Hs.446091	NM_152858;NM_152857;NM_004906	-0.74	-1.52	7.33E-03	6.66E-05
	WTAP	174545	Hs.446091	NM_152858;NM_152857	-1.65	-1.67	1.48E-03	1.39E-03
250	XRN1	107026	Hs.435103	NM_019001;NM_001042604	-0.30	-0.87	6.64E-02	1.79E-04
251	YWHAZ	98307	Hs.492407	NM_145690;NM_003406	-0.68	-1.78	5.39E-05	3.88E-07
252	ZAK	106461	Hs.444451	NM_133646	-1.23	-1.93	2.16E-05	1.15E-06
253	ZDHHC2	124794	Hs.443852	NM_016353	-0.66	-1.52	1.10E-03	8.43E-06
254	ZFAND6	32963	Hs.306329	NM_019006	-0.81	-1.57	2.28E-04	2.74E-06
255	ZFP64	28947	Hs.473082	NM_199426;NM_018197;NM_022088	-0.50	-0.67	1.83E-03	1.45E-04
256	ZMYM4	8883	Hs.269211	NM_005095	-0.41	-1.05	7.35E-04	6.84E-06
257	ZNF238	16779	Hs.69997	NM_006352;NM_205768	-0.32	-0.57	1.48E-02	3.32E-04
258	ZNF294	158	Hs.288773	NM_015565	-0.09	-0.95	6.79E-01	2.28E-04
259	ZNF650	63872	Hs.379548	NM_172070	-0.01	-0.68	9.41E-01	2.71E-05
260	ZNF721	2272	Hs.428360	NM_133474	-0.65	-0.68	6.67E-05	3.49E-05
